# Spatial Reconstruction of Oligo and Single Cells by De Novo Coalescent Embedding of Transcriptomic Networks

**DOI:** 10.1002/advs.202206307

**Published:** 2023-06-15

**Authors:** Yuxuan Zhao, Shiqiang Zhang, Jian Xu, Yangyang Yu, Guangdun Peng, Carlo Vittorio Cannistraci, Jing‐Dong J. Han

**Affiliations:** ^1^ Peking‐Tsinghua Center for Life Sciences Academy for Advanced Interdisciplinary Studies Center for Quantitative Biology (CQB) Peking University Beijing 100871 P. R. China; ^2^ CAS Key Laboratory of Computational Biology Shanghai Institute of Nutrition and Health Chinese Academy of Sciences 320 Yue Yang Road Shanghai 200031 P. R. China; ^3^ Center for Cell Lineage and Development CAS Key Laboratory of Regenerative Biology Guangdong Provincial Key Laboratory of Stem Cell and Regenerative Medicine GIBH‐HKU Guangdong‐Hong Kong Stem Cell and Regenerative Medicine Research Centre Guangzhou Institutes of Biomedicine and Health Chinese Academy of Sciences Guangzhou 510530 P. R. China; ^4^ Center for Cell Lineage and Atlas Bioland Laboratory Guangzhou 510530 P. R. China; ^5^ Biomedical Cybernetics Group, Biotechnology Center (BIOTEC) Center for Molecular and Cellular Bioengineering (CMCB) Technische Universität Dresden Tatzberg 47–49 01307 Dresden Germany; ^6^ Center for Complex Network Intelligence (CCNI) Tsinghua Laboratory of Brain and Intelligence (THBI) Department of Physics, Department of Computer Science Department of Biomedical Engineering Tsinghua University 60 Chengfu Road Beijing 100084 P. R. China; ^7^ Center for Systems Biology Dresden (CSBD) Pfotenhauerstr 108, 01307 Dresden Germany

**Keywords:** coalescent embedding, single cell, spatial reconstruction, spatial gene expression, spatial gene marker

## Abstract

Single cell RNA‐seq (scRNA‐seq) profiles conceal temporal and spatial tissue developmental information. De novo reconstruction of single cell temporal trajectory has been fairly addressed, but reverse engineering single cell 3D spatial tissue organization is hitherto landmark based, and de novo spatial reconstruction is a compelling computational open problem. Here it is shown that a proposed algorithm for de novo coalescent embedding (D‐CE) of oligo/single cell transcriptomic networks can help to address this problem. Relying on the spatial information encoded in the expression patterns of genes, it is found that D‐CE of cell–cell association transcriptomic networks, by preserving mesoscale network organization, captures spatial domains, identifies spatially expressed genes, reconstructs cell samples’ 3D spatial distribution, and uncovers spatial domains and markers necessary for understanding the design principles on spatial organization and pattern formation. Comparison to the novoSpaRC and CSOmap (the only available de novo 3D spatial reconstruction methods) on 14 datasets and 497 reconstructions, reveals a significantly superior performance of D‐CE.

## Graphical abstract

The authors develop a novel de novo oligo and single cells spatial reconstruction method, developmental coalescent embedding (D‐CE). DC‐E can further optimize the reconstruction result by adding a template or markers. Under the evaluation of three indexes, ordering index, angular separation index, and PSImcc, DC‐E shows superior reconstruction performance compared with novoSpaRC, CSOmap, and other three methods.

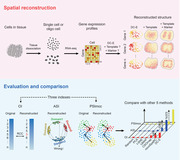

## 1 Introduction

**Figure 1:**
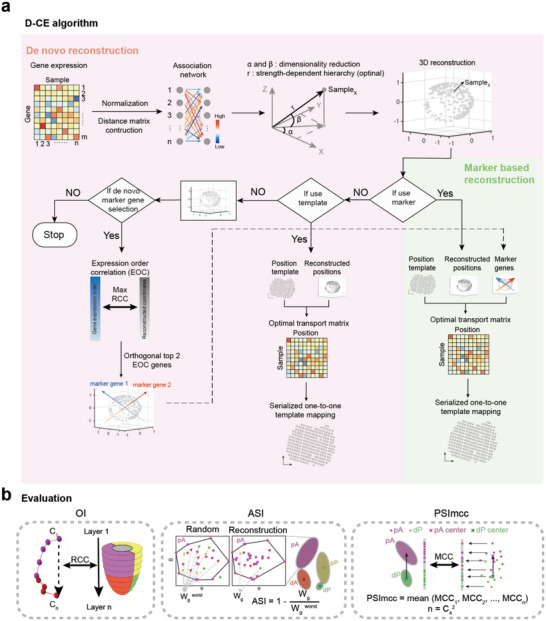
D‐CE method of spatial reconstruction of 3D localizations. a) Overview of spatial structure reconstruction by D‐CE, which consists of angular and radial reconstruction. Angular reconstruction is based on dimensionality reduction by singular value decomposition (SVD), radial reconstruction is based on the strength‐dependent hierarchy. Pearson distance and SQRT (square root of each element of the matrix) were applied to all expressed genes to construct an association network for D‐CE. Maximum EOC (expression order correlation) were calculated for all expressed genes in all directions by rotating the reconstructed structure at 12° per step in two orthogonal directions, respectively. If a position template is available with or without using marker genes, the D‐CE reconstructed coordinates were serially one‐to‐one mapped to the template after optimal transport to D‐CE‐t reconstructions. The top EOC genes on the 2 and 3 orthogonal directions are use as marker gene 1, 2, and 3 for 2D and 3D template fitting, respectively. b) Then ordering index (OI), angular separation index (ASI), and projection separability index of Matthews correlation coefficient (PSImcc) to the original sample layer order were used to assess the accuracy of spatial reconstruction of samples’ positions.

Cell identity transition is precisely controlled and ordered, this implies that individual single cells are genetically fingerprinted and genomically programmed to evolve toward a 3D spatial tissue continuum. Single cell technologies—such as single cell RNA‐seq (scRNA‐seq) that simultaneously profile thousands and more single cells—have becoming powerful tools to capture such continuous spatiotemporal changes during development.^[^ ^1^ ^]^ Based on single cell profiles, the transition paths to the differentiated cells (or the developmental time trajectories) can be reconstructed by calculating transcriptomic similarities or dissimilarities between single cells. Various computational tools based on this assumption have been established to model the developmental time trajectories.^[^ ^2^ ^]^ For instance, Monocle reduces the data dimensionality and uses the minimum spanning tree to model the developmental paths.^[^ ^3^ ^]^ Diffusion pseudotime, which is based on diffusion‐like random walk distances, is used to map developmental branching decisions.^[^ ^4^ ^]^ Spatial distribution and patterning of cells are not only essential for development but also for disease diagnosis and prognosis.^[^ ^5^ ^]^ Patterning and morphogenesis during development, injury or regeneration are processes of cell movements and organizations guided by three major forces: small or macromolecule morphogen gradient, bioelectric voltage gradient and mechanical force gradient, forming local environment or spatial domains, and program or reprogram the cells at the epigenome and transcriptome levels.^[^ ^6^ ^]^ Thus, transcriptomic‐based computational reverse engineering of 3D tissue distribution in a “pseudospace” could be potentially achieved.^[^ ^7^ ^]^ A benchmark cell population timer/clock has been proposed to test the performance of pseudotime algorithms to approximate the real developmental time of each single cell.^[^ ^8^ ^]^ Similarly, a benchmark microdissection‐based 3D transcriptome, termed Geo‐seq,^[^ ^9^ ^]^ can be used to evaluate algorithms to approximately map single cells onto in vivo positions.^[^ ^1^ ^]^ While technologies to generate spatially resolved transcriptomes are still evolving and not yet achieving single cell resolution or in 3D space, the vast majority of the millions perhaps billions of scRNA‐seq can benefit from de novo single cell level 3D reconstruction. Currently, there exist only two algorithms (novoSpaRC^[^ ^10^ ^]^ and CSOmap^[^ ^11^ ^]^) to do so computationally, that is to de novo reconstruct the spatial cell distributions and spatial transcriptomes, with encouraging yet highly variable results. Thus, de novo spatial reconstruction is still a compelling computational open problem.^[^ ^12^ ^]^ Some landmark‐based computational approaches, such as Seurat and Halpern et al.’s method, have been proposed to reconstruct spatial distribution of single cell transcriptomes in zebra fish embryos and mouse liver based on preselected or verified spatially expressed landmark genes.^[^ ^13^ ^]^ But, these approaches are not de novo reconstruction methods because the landmark genes are ad hoc expressed in certain specific 3D spatial positions of the considered in vivo tissue, and act as surrogate labels for the positions when revealed and input to the algorithms. So far, effective, universally (tissue‐wide) applicable, completely de novo approaches have yet to be developed, and only recently the first template structure constrained approach named novoSpaRC was proposed by Nitzan et al.^[^ ^10^ ^]^ with encouraging yet variable results highly dependent on the input template. CSOmap based on ligand–receptor interactions is another de novo method recently developed,^[^ ^11^ ^]^ but performs no better than novoSpaRC.^[^ ^14^ ^]^ To address the de novo reconstruction of oligo and single cell 3D spatial tissue ordering and localization, we design a novel algorithm termed De Novo Coalescent Embedding (D‐CE) according to the network‐based conceptual framework of Coalescent Embedding (CE), which is a model‐free unsupervised machine intelligence methodology for network geometry embedding.^[^ ^15^ ^]^ However, D‐CE is remarkably different from previous CE algorithms, which were designed for hyperbolic embedding.^[^ ^15^ ^]^ Although it shares with them the rationale to exploit the phenomenon of network coalescence,^[^ ^15^ ^]^ D‐CE is innovative because it introduces for the first time a de novo network‐based strategy in spatial pattern reconstruction of oligo and single cells (**Figure** **1**a) and also because, it simultaneously provides a de novo spatial marker gene nomination algorithm for self‐generated markers, which can further guide customizable template‐based reconstruction. The D‐CE algorithm is based on the same principle/assumption of all single cell position mapping methods given gold standard transcriptome of positional landmark samples, that is, the single cells or oligo cells from the same spatial position/domain have similar transcriptome profiles, which have been demonstrated in our and others’ previous works,^[^ ^1^, ^9^, ^16^ ^]^ except that spatial position/domain here is the hidden variables in our algorithm, while they are the input variables the mapping algorithms.

## 2 Results

### 2.1 De Novo Coalescent Embedding (D‐CE): A Novel Network‐Based Algorithm for Cells Spatial Pattern Reconstruction

CE encloses under its name a class of machine intelligence algorithms for efficient embedding of large real networks to the latent geometric space, which have been proven to impact hyperbolic big‐network‐data analysis in biology, neuroscience, and social science.^[^ ^15^ ^]^ For instance, CE showed to boost the detection of community structural organization in social networks^[^ ^15^ ^]^ and to reliably capture the original geometry of macroscale structural brain connectomes.^[^ ^17^ ^]^ The name coalescent embedding derives from “angular coalescence,” which is a term proposed to indicate that, as a result of this methodology of embedding, the individual network nodes geometrically aggregate together (from the Latin verb coalēscō: to join, merge, amalgamate single elements into a single mass or pattern) forming a pattern that is progressively ordered along the geometrical angular coordinates.^[^ ^15^ ^]^ In CE algorithms, the node angular coordinates are ordered according to latent relations of topological homophily (similarity) between the network nodes,^[^ ^15^ ^]^ instead the node radial coordinates according to latent relations of topological hierarchy between the network nodes^[^ ^15^ ^]^ (Figure 1a). Our core assumption in D‐CE, in line with the literature in this field,^[^ ^6a,d^ ^]^ is that local environment programs the transcriptome to form discernable local cell–cell network neighborhood, and our innovation is that the D‐CE cell–cell network embedding captures co‐occurrent transcriptomic relation between the cells in the local environment and map their geometrical relations within and in between accordingly.

The network embedding step, is designed according to topological machine learning theory,^[^ ^18^ ^]^ and it aims for the preservation of the shortest‐path network connectivity^[^ ^18^ ^]^ in the 3D reconstruct space by means of the singular value decomposition (SVD) decomposition of the centered shortest‐path kernel obtained from the transcriptome‐similarity network. The result of this network embedding procedure is the angular coalescence^[^ ^15^ ^]^ that is an angular similarity pattern which preserves the mesoscale network structure of cell‐assemblies (which are network communities: cohort of cells with higher intrasimilarity in respect to the rest of the network) and the relationships among cell‐assemblies, and these relationships map the relative locations of the cell‐assemblies and entities within.

There is so far no genome wide single cell 3D spatial transcriptomics technology or datasets exist, which underscores the urgent need to develop accurate computational de novo reconstruction methods. As Geo‐seq is the only 3D genome‐wide oligo cell spatial transcriptome with known ground‐truth spatial domain labels,^[^ ^1^, ^9^ ^]^ in this study, we first use this dataset to establish our 3D reconstruction D‐CE method (Figure 1). We then validate D‐CE on other independent 2D or 3D oligo cell or single cell transcriptomic datasets that contain gold standard labels at either the spatial domain level (from microdissection) or at template level (from 1D or 2D genome‐wide spatial transcriptomes or 3D imaging of a small number of spatially distributed genes, where each sample's position on a template is known) (Figure 1a). As spatial templates are known for the template level datasets, we further developed a template constrained D‐CE (D‐CE‐t) to allow the fitting of D‐CE embedding to an existing or customized spatial template (Figure 1a). This is done by first solving an optimal transport problem between samples and D‐CE reconstructed positions, then one‐to‐one mapping each sample to a specific spatial position on the template.^[^ ^19^ ^]^

Additionally, D‐CE also uncovers top spatially distributed genes (some might be potential morphogens) as marker genes. The expression patterns of these self‐nominated marker genes, when used, can further enhance the accuracy of spatial template‐fitting. This is no longer a mere de novo reconstruction, but rather a self‐generated (because the makers are self‐generated by D‐CE and not indicated by previous knowledge in scientific literature) marker‐based reconstruction (Figure 1a). The introduction of a self‐generated marked‐based spatial reconstruction represents another important computational innovation of our study, and a major advance in the field. This is done by selecting the top spatially expressed marker genes at the two predominant but orthogonal spatial orientations according to the correlation between gene expression level and the spatial order of the samples (Expression Order Correlation, EOC). When the expression levels of the marker genes at each position of the template is revealed to the mapping algorithm, the expression levels of the markers are used to weight the cost to transport from each sample to each position^[^ ^19^ ^]^ (Figure 1a). In fact, due to the importance of spatial marker genes, algorithms such as Trendsceek^[^ ^20^ ^]^ were developed to specifically search for spatially expressed genes, which can also be used as markers in this step depending on users’ preference.

### 2.2 Testing D‐CE on Domain Annotated Oligo and Single Cell RNA‐seq Data

Data normalization and distance metric exploration are routine preprocessing steps in computational biology.^[^ ^21^ ^]^ We investigated the impact of different normalization strategies and distance metrics on the performance of the considered algorithms (our proposed and the ones used for comparison), which are then fairly compared by considering the best of these settings. In order to appraise the extent to which different normalization methods and distance metrics would affect the performance of D‐CE, we considered Geo‐seq samples^[^ ^9^ ^]^ whose 3D domain labels are known and can be used as gold standards to evaluate the reliability of a de novo 3D reconstruction algorithm. Each Geo‐seq sample is not a single cell but a portion of tissue constituted by a cohort of ≈10–20 single cells, however this genome‐wide 3D labeled dataset is an ideal dataset to design and to test D‐CE performance. We considered the sample–sample transcriptome distance matrix (weighted network) using each of 12 normalization methods (Table S1, Supporting Information) and each of 5 distance measures (Figure 1a), resulting in a combination of candidate association networks to test, and embedded each of them separately using the proposed D‐CE algorithm (Figure 1a, Experimental Section). Specifically, we considered 5 distance measures, including: Spearman distance^[^ ^22^ ^]^ (1‐Spearman rank coefficient (RCC)), Pearson distance (PD) (1‐Pearson correlation coefficient (PCC)), Euclidean distance (ED), and PCC and RCC filtered by connectivity specificity index (CSI),^[^ ^23^ ^]^ named PCC‐CSI and RCC‐CSI (Figure 1a and Experimental Section; and Figure S1, Supporting Information).

**Figure 2:**
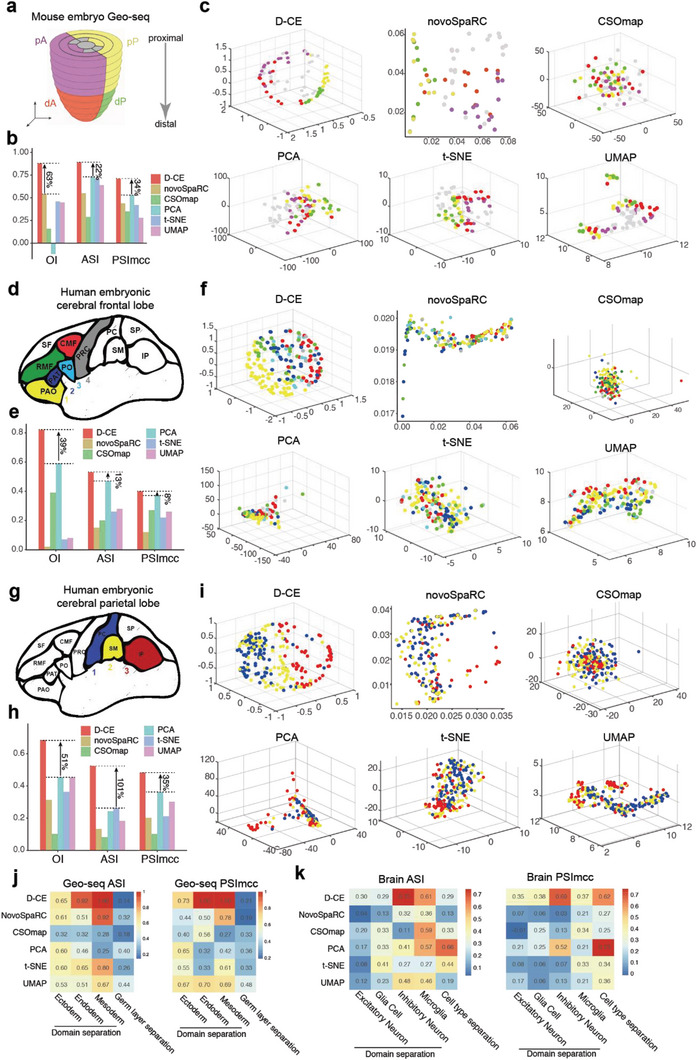
Reconstruction of spatial domain labels from oligo or single cell RNA‐seq data with by D‐CE, novoSpaRC, CSOmap, PCA, t‐SNE, and UMAP. a) Illustration of 4 spatial domains pA, dA, pP, and dP in mouse embryo E7.5. b) Barplot of OI, ASI, and PSImcc for D‐CE, novoSpaRC, CSOmap, PCA, t‐SNE, and UMAP reconstructions. c) Reconstructed structure of D‐CE, novoSpaRC, CSOmap, PCA, t‐SNE, and UMAP. d–f) The same layout as panel (a) to (c) for human embryonic frontal lobe. Six regions from frontal lobe, CMF (caudal‐middle‐frontal), PRC (precentral), PAO (pars orbitalis), PO (pars opercularis), RMF (rostral‐middle‐frontal), and PAT (pars triangulars), are used as domain labels of the scRNA‐seq data. As only microglia cells contain >3 cells per region, they are the only cell type used for reconstruction. OI is calculated between the known domain order and the reconstructed coordinates of PAO, PAT, PO, and PRC (ordered from 1–4). g–i) The same layout as panel (a–c) for human embryonic parietal lobe. 3 regions from parietal lobe (PC (postcentral), SM (supra‐maginal), and IP (inferior parietal)) as domain labels. OI is calculated between the known order and the reconstructed coordinates of PC, SM, and IP (ordered from 1 to 3). For panels (b, e, and h) the percentage of improvement by D‐CE over the second‐best method is labeled for each index. j) ASI (left panel) and PSImcc (right panel) for spatial domain separation and reconstruction of Geo‐seq samples from the same germ layer, as indicated (≈1–3 columns), or for the separation of different germ layers (the last column) by each reconstruction method. k) ASI (left panel) and PSImcc (right panel) for spatial domain separation and reconstruction of single cells in the brain parietal region scRNA‐seq data in panel (g), within each cell type that contains >3 cells per region, as indicated (≈1–4 columns), or the separation of different cell types (the last column).

We tested each of these 60 D‐CE candidate strategies on a number of gene sets, including all expressed genes, or annotated developmental, signaling genes, and transcription factors, to spatially reconstruct the Geo‐seq data of different germ layers in mouse early embryo development gastrulation stage (E6.5, E7.0, and E7.5). To evaluate the accuracy of the reconstruction, we grouped the Geo‐seq samples into four groups: 1–2) proximal anterior and distal anterior (pA and dA); 3–4) proximal posterior and distal posterior (pP and dP) (Figure 1b). Angular separation index^[^ ^15^ ^]^ (ASI, ranging from 0 to 1 with 1 being perfect separation, see the Experimental Section for details) is used to test how well the four groups are separated according to angular coordinates in the embedding space. Projection separability index‐Matthews correlation coefficient (PSImcc, ranging from 0 to 1 with 1 being perfect separation on the optimal projection line, Experimental Section) is used to evaluate the group separability and relative orientation in geometrical space. Ordering Index (OI, ranging from −1 to 1 with 1 being perfect agreement between the original order and the reconstructed order) to evaluate the accuracy of ordering the layers from distal to proximal (e.g., layer 1–11 in E7.0 embryo, Figure 1b and Experimental Section). Finally, to evaluate with one unique value for each of the 60 embedding strategies on various gene sets (Figure S1, Supporting Information), we consider the maximum rank of these three indices for each embedding,^[^ ^19^ ^]^ this means that a method that ranks 1 for each of these three indices will be the perfect candidate to be selected, because it has the lowest maximum ranking across the three possible indices. Indeed, the lowest maximum rank will indicate the best reconstruction of both the anterior and posterior localization and the order of layers (Figure S2g, Supporting Information), and the best strategy to perform D‐CE turns out to be based on square root of Pearson distance for the network construction, which works especially well on all expressed genes, and works best on the intersection of developmental genes, signaling genes, and transcription factor genes with a total of 639 genes. The D‐CE reconstruction showed a very high correspondence to the original geometric domains of the samples in the mouse embryo from where the Geo‐seq samples were derived (**Figure** **2**a–c). The anterior and posterior samples of different germ layers in different stages are well separated into the opposing directions of the 3D space, similar to its original distribution in the developing mouse embryo, so are the proximal and distal samples, as evidenced by the high ASI separating the pA, dA, pP, and dP samples with all germ layers combined together at stage E6.5, E7.0, and E7.5, or each germ layer separately (Figure 2a–c; and Figure S2a–f, Supporting Information). In the reconstructed structure, samples are not separated by different germ layers, indicating that the samples are not distributed according to lineage (Figure S3, Supporting Information). That the left and right samples of the same layers are aggregated together (Figure 2a) is expected because they are highly symmetric and have no expression differences as previously observed.^[^ ^1^ ^]^

After evaluating the D‐CE algorithm on Geo‐seq data, we test D‐CE on other datasets. Apparently, cell type labels cannot be used as spatial or position labels, as cells of the same cell type often distributed in very different spatial domains, and one spatial domain often contain many different cell types. For this reason, we confined the testing of reconstruction to only two types of data, one is the oligo or single cells microdissected from spatial domains, thus have definitive domain labels, and two is the template‐based spatial transcriptomes at oligo or single cell resolution, with definitive position label for each sample in a spatial template (Table S2, Supporting Information). These result in 14 datasets and 497 reconstructions: 43% (6 in 14) of the datasets or 8% of the reconstructions (39 in 497) are single cell data, as shown in Table S2 (Supporting Information), the rest are spatial transcriptome samples consisting of 10–40 cells per sample (Table S2, Supporting Information).

We first examined whether D‐CE can be applied to three scRNA‐seq datasets with annotated domain labels, that is, scRNA‐seq of spatial domain microdissected samples. The human embryonic brain frontal and parietal lobe scRNA‐seq datasets contain the brain region labels from which the single cells are dissected (Figure 2d,g), we thus used these labels as ground‐truth domain labels. As the annotated ordering of the regions is a bit artificial, to unbiasedly examine these datasets, we examined whether our reconstruction can reveal more spatially expressed genes than the annotated ordering, i.e., the relative 3D locations of several brain regions. Indeed, D‐CE and NovoSpaRC reconstructed structures both give higher EOC values to gene expressions than the simple artificially annotated ordering (Figure S4a, Supporting Information). Interestingly, only the D‐CE reconstructed EOC marker genes show an enrichment for oxygen binding, but not the manually labeled order related EOC correlated genes (Figure S4b,c, Supporting Information). Oxygen gradient is known to play a role in directing cell differentiation and pattern formation, including the brain pattern.^[^ ^24^ ^]^ This biological finding further demonstrates the necessity of a reliable reconstruction method, which is even more accurate than manual labeling and annotations. D‐CE could still accurately distinguish spatial orders when brain frontal and parietal lobe samples are combined, with its superior performance more distinguished form the other methods than for frontal lobe alone, but less than parietal lobe alone (Figure S5, Supporting Information).

Using ASI, OI, and PSImcc as performance measures on the Geo‐seq data and the domain labeled scRNA‐seq data, we compared D‐CE versus 5 methods: 3D‐PCA, 3D‐tSNE, 3D‐UMAP that are 3 state‐of‐the art dimensionality reduction methods; and NovoSpaRC^[^ ^10^ ^]^ and CSOmap^[^ ^11^ ^]^ that are the only two existing de novo reconstruction methods. Apparently, NovoSpaRC and CSOmap rarely perform better than the 3 popular dimensionality reductions methods, whereas D‐CE always performs the best whether compared to these de novo reconstruction methods or the dimensionality reduction methods (Figure 2b,e,h). The advantage of D‐CE over the other methods can be also easily observed on the microsurgical mouse brain oligo cell RNA‐seq dataset with 1D domain labels, each corresponding to one cortex layer (Figure S6, Supporting Information).

To understand why the D‐CE is superior to other methods for spatial reconstruction, we investigated why other methods failed. One possible reason is that based on sample–sample similarities only, the other methods are unable to distinguish similarity as a result of lineage, or as a result of responding to common regionalization or patterning cues, which in turn might be encoded in the nonlinear manifold similarities estimated by a network‐driven approach such as D‐CE. Indeed, for the Geo‐seq data—where samples are known for both their spatial domains and germ layers—only D‐CE can well separate and reconstruct spatial positions of samples from the same germ layer (lineage), while other methods, in particular PCA and UMAP mostly separate samples based on germ layers rather than spatial domains (Figure 2j). The fact that standard linear and nonlinear dimension reduction methods (whose category includes PCA and UMAP) mainly separate transcriptomic samples by germ layers was already discovered in our past studies,^[^ ^9^, ^22^ ^]^ therefore our results are reliable and represent a further confirmation of this evidence. However, the relevant finding that D‐CE is the only method able to discover both spatial and germ layer information suggests that the first D‐CE step is indeed able, by network‐reconstruction, to better capture the latent transcriptomic manifold modes of the complex biosystem that generates the data.

Similarly, for the mouse brain parietal region scRNA‐seq data, for which both the brain domain labels and the cell type labels are known, only D‐CE can well separate and reconstruct spatial positions of single cells from the same cell type (lineage), while other methods, in particular PCA and t‐SNE mostly separate samples based on cell types rather than spatial domains (Figure 2k). These biological evidences gained by D‐CE support the notion that cell–cell similarities (like trait similarity between human individuals) are a result of both lineage and spatial environment effects, and highlights the importance of appropriate network embedding instead of mere dimension reduction to decipher the local connectivity and neighborhood structure for spatial reconstructions.

### 2.3 Testing D‐CE on Template‐Based Position Labeled Oligo and Single Cell RNA‐seq Data

Next, we investigated whether D‐CE is also applicable to other spatial transcriptomic data with gold standard position labels in a template for each sample, either at oligo cell or single cell resolutions.

We first compared our spatial reconstruction with novoSpaRC on the 3D gene expression dataset BDTNP,^[^ ^25^ ^]^ on which was novoSpaRC developed and optimized. As only the expression data of 84 Drosophila embryo development regulating TFs are measured (no ligand and receptor genes), CSOmap, which only accepts human or mouse data and using ligand and receptor genes for reconstruction, is not applicable on this dataset. All the 84 genes were used for D‐CE reconstruction. We found that, instead of PD (as before), its local‐threshold‐variation named PCC‐CSI (which is a distance obtained by local threshold of Pearson correlation using CSI) performs the best for spatial reconstruction among all 5 distance options (Figure S7, Supporting Information). This difference in comparison to the Geo‐seq data might be attributed to the large number of nodes in this network (3039 in BDTNP vs <100 in Geo‐seq and other domain labeled datasets). A gradient down‐sampling of the BDTNP to data indeed shows that PD performs better than PCC‐CSI when the sample number is <150, beyond that PCC‐CSI performs better as shown by ASI and PSImcc (Figure S7, Supporting Information). OI is not evaluated because in the down‐sampling, samples are randomly selected and hence rarely from a straight line along the *x*, *y*, or *z* axis, and this disrupts the original distribution of the samples on 3 axes, making the OI inapplicable to the randomly sampled networks for evaluating the spatial reconstruction of down‐sampled samples.

**Figure 3:**
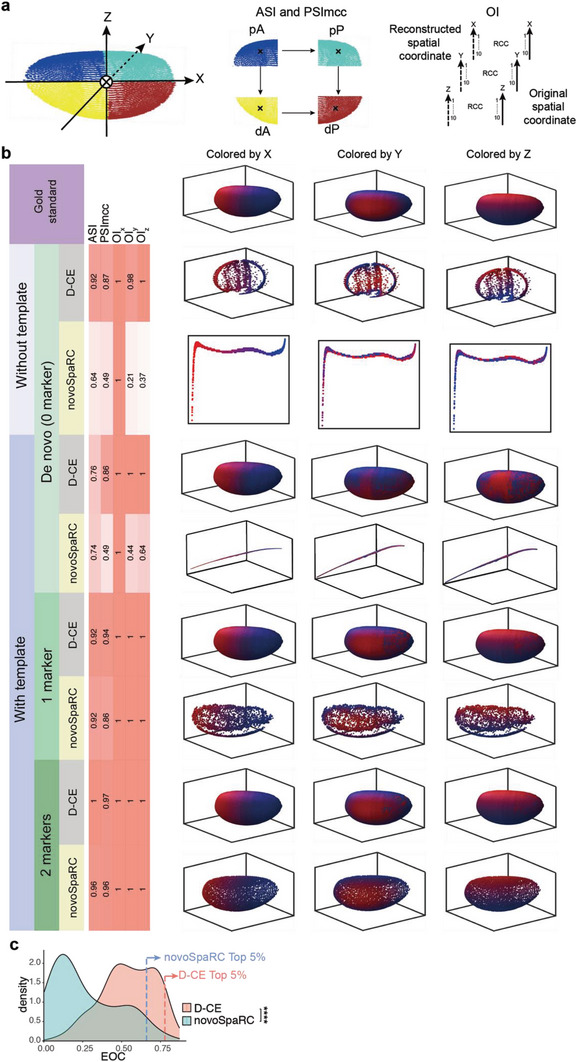
Comparison of spatial reconstruction of BDTNP dataset using D‐CE and novoSpaRC. a) Illustration of Drosophila embryo segmentation for ASI and PSImcc and the OI*_x_*, OI*_y_*, and OI*_z_* calculation. The embryo was divided into 4 groups along the x and z coordinates (a, middle) by spatial locations for ASI and PSImcc calculation. Each coordinate was sorted and divided into 10 groups (a, right) and OI was calculated based on the gold standard original spatial coordinate. b, Original spatial positions (row 1) in Drosophila embryo examined by the BDTNP dataset, which is colored by spatial coordinates on *x* (left), *y* (middle), and *z*‐axis,^[^ ^42^ ^]^ respectively. Reference coordinates of *x*, *y*, and *z* axis are labeled in ascending order with a color gradient from blue to red, which is also used to paint the samples in the reconstructed structures to visualize their 3D orders in the following panels. D‐CE and novoSpaRC spatial reconstruction of BDTNP (row 2 and 3). D‐CE‐t and NovoSpaRC spatial reconstruction of BDTNP dataset with 0 marker (row 4 and 5), 1 marker (row 6 and 7) and 2 markers (row 8 and 9) visualized by sample color code designated by the gold standards (top panel) for X, Y, and Z axis, respectively. Indexes of spatial reconstruction evaluation are shown as a column scaled heatmap next to each reconstructed embryo. NovoSpaRC randomly selects 1 or 2 markers for marker‐based template fitting, the best result among 100 trials is used for novoSpaRC. c, Density plot of all expressed genes to the D‐CE and novoSpaRC reconstructions’ coordinates. The dashed line indicates the EOC position of top 5% genes in each distribution. Student's *t*‐test was used to compare the distribution difference between D‐CE and the other two methods. ****p‐value < 2.2e‐16.

D‐CE reconstructed spatial order is highly similar to the original 3D coordinates on the embryos structure (**Figure** **3**a,b). For novoSpaRC, using the dot product of the optimal sample and location probabilistic coupling matrix *T*_*m* × *n*_ inferred by novoSpaRC and the original location *L*_*n* × 3_ (position template) as the reconstructed locations for each sample,^[^ ^19^ ^]^ we visualized its spatial reconstructions based on 0, top 1 or top 2 marker genes used. Consistent with the visual appearances, judged by the quantitative parameters of OI, ASI, and PSImcc, the de novo reconstruction by D‐CE is much closer to the annotated original gold standard structure than that by NovoSpaRC (Figure 3b), and have overall higher EOCs among all input genes or the top 5% EOC genes (Figure 3c). The spatial marker genes identified by D‐CE according to EOC, indeed recovered well‐known spatially expressed genes in the fly embryo, such as Ilp4 (top marker 1) and tsh (top marker 2) (Figure 3b). These marker genes can be further used as references to refine the reconstruction, in which case the reconstruction is no longer de novo, but self‐generated marker based (Figure 3b). Using D‐CE's one to one positioned template mapping (D‐CE‐t), we observed the template‐fitted reconstruction even without any marker genes (D‐CE‐t 0 marker, still de novo) can reconstruct the sample orders on the *x*‐axis, although fitting to the *y* and *z* axis seems to require markers on these dimensions. In fact, with only one marker (top 1 marker) identified by D‐CE allows D‐CE‐t fully reconstruct the sample distribution on all 3 dimensions (Figure 3b). These results are further confirmed by the expression patterns of 4 spatially distributed TF genes, sna, Kr, eve, and ken (Figure S8, Supporting Information). Visually without markers, D‐CE is much better than the novoSpaRc, and when 1 or 2 markers are used, they both look good (Figure S8a, Supporting Information), but when more precisely quantified by OI, D‐CE‐t is better than novoSpaRc with 0, 1, and 2 markers (Figure S8b, Supporting Information). D‐CE completely reconstructed the ventral expression pattern of the sna gene and the vertical bi‐stripe pattern of Kr, recovered 6 out of 7 strips of eve and both stripes of ken with nonperfect placement. Whereas the de novo novoSpaRC without any marker gene only partially reconstructed the pattern of ken, but completely failed to recover the sna expression pattern, wrongly aggregated 7 stripes of eve into one broad stripe and recovered one of the two stripes of Kr (Figure S9, Supporting Information). It should be noted that novoSpaRC by default only provides a 2D‐grid template, thus only 2D reconstruction can be performed, and it selects markers randomly, so for a favorable comparison for novoSpaRC, we used the best among 100 random marker‐based reconstruction results to represent its performance (Figure 2b; and Figure S10, Supporting Information).

To further test whether D‐CE can reconstruct spatial gene expression patterns of different cells directly using scRNA‐seq data, we applied it to a Drosophila embryo scRNA‐seq dataset^[^ ^10^ ^]^ (Figure S9, Supporting Information) and a zebrafish embryo blastoderm cap scRNA‐seq dataset^[^ ^13a^ ^]^ (Figure S10, Supporting Information). In the reconstructed Drosophila embryo, the expression pattern of dorsal/ventral specific gene (such as ush, twi, and sna) are highly correlated with the FISH images downloaded from BDGP^[^ ^26^ ^]^ dataset, with OI > 0.5. For anterior/posterior specific genes (such as ImpE2 and Adgf‐1), the pattern is not as good as dorsal/ventral pattern. For zebrafish embryo blastoderm cap, D‐CE well reconstructed the dome shape of the blastoderm cap, while NovoSpaRC only captured the top outline (Figure S10, Supporting Information). The gene expression patterns of 9 spatial specific genes show high correlation (OI > 0.5) with the FISH images (downloaded from the ZFIN database^[^ ^27^ ^]^). Compared with novoSpaRC, D‐CE reconstructed gene expression patterns have significantly higher OI to the FISH images (Wilcoxon signed rank test *p* = 0.03 and 0.04 for the 6 and 9 spatially expressed Drosophila and zebrafish genes previously tested^[^ ^10^ ^]^). Again, as CSOmap accepts only human or mouse data as input, is not applicable to datasets of other species.

**Figure 4:**
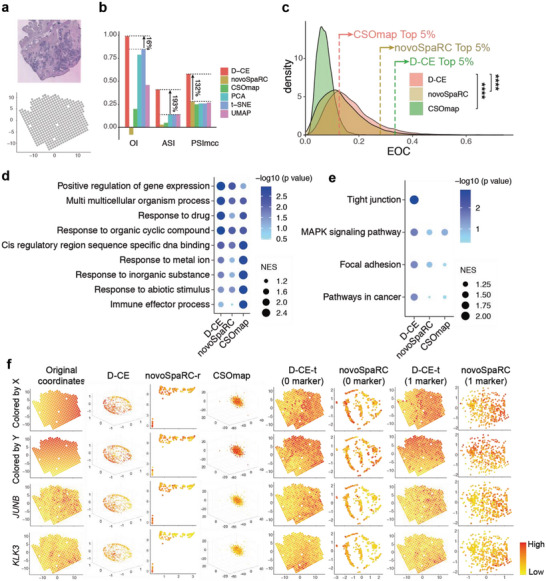
Spatial reconstruction and spatial marker gene detection of cancerous prostate spatial transcriptomic dataset with D‐CE, novoSpaRC, CSOmap, PCA, t‐SNE, and UMAP. a) Illustration of a cancerous prostate transcriptomic data. Tissue staining picture reproduced from Berglund, E. et al. (upper panel) and original coordinates of all samples on a template (lower panel). b) Barplot of OI, ASI and PSImcc. The percentage of improvement by D‐CE over the second‐best method is labeled for each index. c) Density plot of all expressed genes to the reconstructed coordinates by D‐CE, novoSpaRC, and CSOmap. The dashed line indicates the EOC position of top 5% genes in each distribution. Student's *t*‐test was used to compare the distribution difference between D‐CE and the other two methods. d,e) GSEA analysis of GO d) and KEGG e) enrichment terms of top 5% EOC genes in panel (c). f) Original coordinates, D‐CE, novoSpaRC, and CSOmap without marker and template fitting, D‐CE‐t with 0 marker, novoSpaRC with template fitting and 0 marker, D‐CE‐t with 1 marker, and novoSpaRC with 1 marker from the first to the eighth column reconstructed coordinates colored according to the X axis, Y axis, and the top two D‐CE markers’ (JUNB and KLK3) expression level from the first to the fourth row, respectively. NovoSpaRC randomly selects 1 or 2 markers for marker‐based template fitting, the best result among 100 trials is used for novoSpaRC. **** *p*‐value < 2.2e‐16.

One of the human spatial 2D template‐based datasets is cancerous prostate spatial transcriptome. For 2D tissue section spatial transcriptome datasets like the cancerous prostate dataset, where annotated domain labels are not available, four artificial domains obtained by dividing the samples at the midpoints of *x* and *y* coordinates are used as golden standard. The de novo reconstruction not only revealed the best performance by D‐CE based on OI, ASI, and PSImcc (**Figure** **4**a,b), but also higher EOCs in general and among the top 5% EOC genes compared to the other two methods (Figure 4c). The biological processes and pathways enriched among the top EOC genes from D‐CE reconstructions are very different from CSOmap, but similar to those from NovoSpaRC, except with higher level of enrichment (Figure 4d,e). Yet only D‐CE, but not NovoSpaRC and CSOmap identified the tight junction that are known to play key roles in spatial patterning^[^ ^6a,d^ ^]^ (Figure 4d,e). As D‐CE and CSOmap are template‐free algorithm for spatial reconstruction, for better comparison, we use a rectangle grid for novoSpaRc reconstruction (novoSpaRc‐r). Since D‐CE‐t does use template information, we compare it directly to novoSpaRc, instead of novoSpaRC‐r. Using the top 1 or 2 EOC genes in orthogonal orientations as markers, further allow for a better marker‐based template fitting by D‐CE‐t (Figure 4f). These marker genes indeed are highly spatially expressed and show distribution gradients in orthogonal directions as predicted by D‐CE (Figure 4f). Remarkably, even without using marker gene, 2D‐template fitted D‐CE (D‐CE‐t) reconstruction (still de novo), nearly fully recapitulated all samples’ spatial distributions in both *x* and *y* dimensions, as well as the marker gene expressions (Figure 4f), and the D‐CE‐t reconstruction with 1 self‐generated marker (JUNB) is almost indistinguishable from the original annotated gold standard structure (Figure 4f). In contrast, novoSpaRC failed to do so without marker, while improved with 1 marker, still cannot reach the level of de novo D‐CE‐t reconstruction (no marker) (Figure 4f). Similar reconstruction performance differences are also found on the human breast cancer dataset (Figure S11a,b, Supporting Information). The EOC distributions of novoSpaRC and D‐CE are very consistent, both are far better than CSOmap (Figure S11c, Supporting Information). While enriched GO terms are similar among the 3 methods, only D‐CE detected an enrichment for KEGG focal adhesion pathway (Figure S11d, Supporting Information). These results further demonstrate the need for a reliable spatial reconstruction to discover spatial domain and markers.

**Figure 5:**
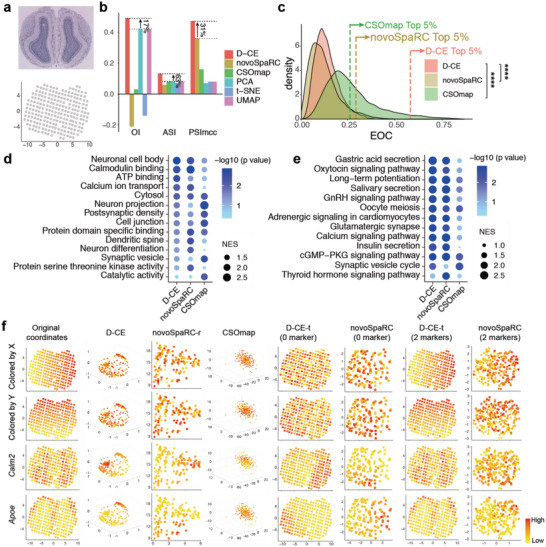
Spatial reconstruction and spatial marker gene detection of mouse olfactory bulb spatial transcriptomic dataset with D‐CE, novoSpaRC, CSOmap, PCA, t‐SNE, and UMAP. a) Illustration of the mouse olfactory bulb spatial transcriptomic data. Tissue staining picture reproduced from Berglund, E. et al. (upper panel) and original coordinates of all samples (lower panel). b) Barplot of OI, ASI and PSImcc. The percentage of improvement by D‐CE over the second‐best method is labeled for each index. c) Density plot of all expressed genes to the reconstructed coordinates by D‐CE, novoSpaRC, and CSOmap. The dashed line indicates the EOC position of top 5% genes in each distribution. Student's *t*‐test was used to compare the difference between D‐CE and the other two methods. d,e) GSEA analysis of GO d) and KEGG e) enrichment terms of top 5% EOC genes in panel (c). f) Original coordinates, D‐CE, novoSpaRC, and CSOmap without marker and template fitting, D‐CE‐t with 0 marker, novoSpaRC with template fitting and 0 marker, D‐CE‐t with 2 marker, and novoSpaRC with 2 marker from the first to the eighth column reconstructed coordinates colored according to the X axis, Y axis, and the top two D‐CE markers’ (Calm2 and Apoe) expression level from the first to the fourth row, respectively. NovoSpaRC randomly selects 1 or 2 markers for marker‐based template fitting, the best result among 100 trials is used for novoSpaRC.

Similarly, the de novo reconstruction of mouse olfactory bulb spatial transcriptome also revealed the best performance by D‐CE based on OI, ASI, and PSImcc (**Figure** **5**a,b), and higher EOC among all genes or the top 5% EOC genes compared to the other two methods (Figure 5c). The biological processes and pathways enriched among the top EOC genes are very similar between D‐CE and NovoSpaRC but rather different from CSOmap (Figure 5d,e). D‐CE identified “neuronal cell body” and “calmodulin binding” ranked as the top pathways (Figure 5d). Indeed, the top two D‐CE EOC genes, Apoe, and Calm2, belong to these two pathways, respectively, and are highly spatially expressed and show distribution gradients in orthogonal directions as predicted by D‐CE (Figure 5f). It should be noted that no de novo method is expected to resolve symmetry in a sample, such as in this olfactory bulb dataset. Thus, not surprisingly, in order to fully reconstruct the symmetric structures, two top orthogonal marker genes identified by de novo D‐CE are needed to guide template fitted reconstruction (Figure 5f). In fact, D‐CE‐t reconstruction with 2 self‐generated markers are nearly identical to the original annotated gold standard structure, so are the marker gene expressions. On the other hand, NovoSpaRC reconstructions with no marker or even 2 markers are far from the original structure, while no obvious pattern can be seen from CSOmap reconstruction (Figure 5f). The primary components (up to 10) the dimensionality reduction approaches (PCA, t‐SNE, and UMAP) are not very informative on spatial ordering, making these approaches not prioritized for spatial reconstruction (Figure S12, Supporting Information).

We find the OI, ASI, and PSImcc of D‐CE reconstructions can still increase with increasing number of markers in both a low reconstruction accuracy (olfactory bulb) and a high reconstruction accuracy (cancerous prostate) dataset, despite at different rate of increase (Figure S13, Supporting Information).

### 2.4 Leap in Performance of D‐CE over Existing Reconstruction Methods

**Figure 6:**
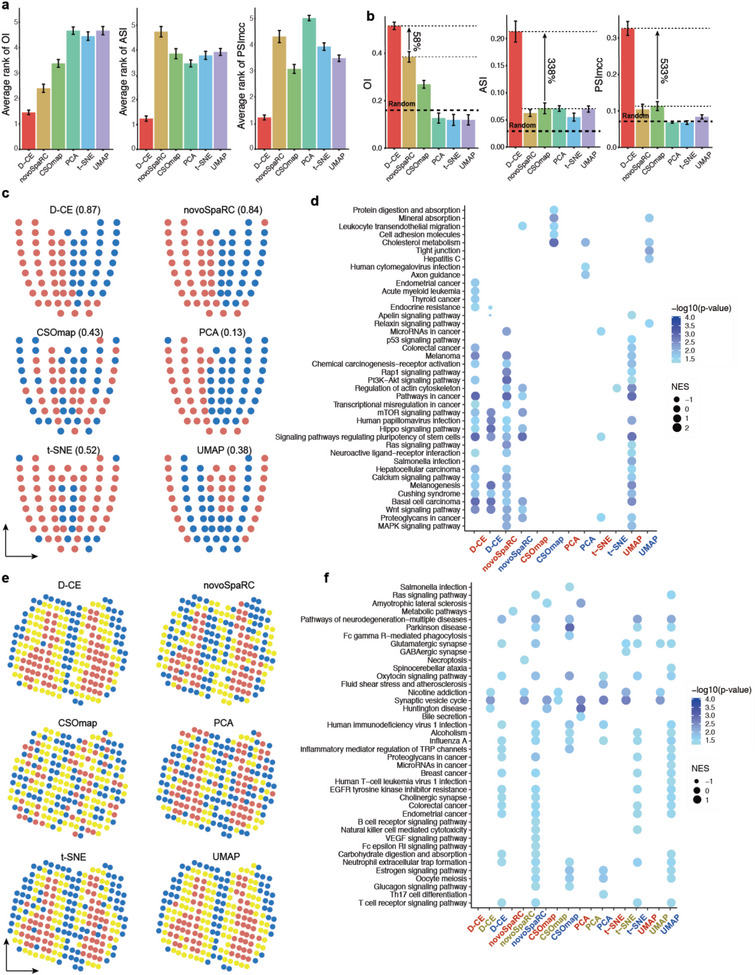
Performance summary and comparison to existing methods and organization and makers of D‐CE into spatial domains. a,b) Average rank g) and values h) of OI, ASI and PSImcc by D‐CE, NovoSpaRC, CSOmap, PCA, t‐SNE, and UMAP on 484 reconstructions from 10 datasets that contain precise sample coordinates on templates. The best performance is ranked 1. Whiskers denote standard deviations. OI is transformed by (1+index)/2 to render all the indexes with a range from 0 to 1. Then each index is normalized by subtracting a baseline value obtained by the average of 100 label‐randomization of the samples. CSOmap is only applicable to 9 datasets and 483 reconstructions (without BDTNP dataset) due to its limit to human and mouse data and LR genes only. Student's *t*‐test *p*‐value are labeled on top. The percentage of improvement by D‐CE over the second‐best method is labeled for each index. c) Domain structures and compositions of mouse embryo Geo‐seq data revealed by D‐CE, novoSpaRC, CSOmap, PCA, t‐SNE, and UMAP. The reconstructed coordinates are clustered by k‐means (*k* = 2), visualized by 2 different colors on the illustrative “corn plot.” Jaccard Index of the two clusters to A and P domains are labeled within the brackets. d, GSEA of KEGG pathways for top 500 cluster specifically up‐regulated genes in all 6 methods. e,f) The same layout as shown in panel (c) and (d) for mouse olfactory bulb data. The reconstructed coordinates are clustered by *k*‐means (*k* = 3) and visualized by 3 different colors on the template.

For a more comprehensive comparison of D‐CE with the existing de novo spatial reconstruction methods novoSpaRC and CSOmap, as well as the state of art dimensionality reduction methods PCA, t‐SNE, and UMAP, we applied them to 6 additional transcriptome datasets with annotated spatial coordinates on a template.^[^ ^5^, ^25^, ^28^ ^]^ For the sake of fairness to all the algorithms for spatial reconstruction, all expressed genes are used during the comparison. The datasets include the melanoma lymph node data,^[^ ^28c^ ^]^ the mouse hippocampus^[^ ^28a^ ^]^ seqFISH data, the mouse brain,^[^ ^28e^ ^]^ seqFISH data, and the mouse medial ganglionic eminence LCM‐seq dataset,^[^ ^28f^ ^]^ the mouse embryonic brain digitized in situ hybridization (ISH) (Figure S14, Supporting Information) and the mouse spinal cord dataset^[^ ^28d^ ^]^ (Figure S15, Supporting Information). Together with the datasets tested above, in total 14 datasets (Table S2, Supporting Information), and 497 reconstructions (one dataset may contain multiple experiments, e.g., barcoded‐microarray based spatial transcriptomic dataset contains 407 arrays) (Table S2, Supporting Information). For example, on the 407 arrays of the mouse spinal cord dataset,^[^ ^28d^ ^]^ D‐CE performs better in the great majority of the 407 reconstructions as evaluated by OI, ASI, and PSImcc, and also by the EOC distribution and the top EOC genes enriched GO/KEGG terms (Figure S15, Supporting Information). Based on the random expectation normalized,^[^ ^19^ ^]^ OI, ASI, and PSImcc on 484 reconstructions from 10 datasets that contain precise sample coordinates on templates, D‐CE ranks the top 1 on all three metrics (**Figure** **6**a), and has an average at least >4‐fold improvement on spatial reconstruction over NovoSpaRC and CSOmap, and three conventional dimensionality reduction methods (PCA, t‐SNE, and UMAP) (Figure 6b). The ASI and PSImcc in particular evidence the unique ability of D‐CE to excel in separating and maintaining homogeneity of spatial domains compared to all other methods. This puts D‐CE at a unique advantage of deciphering the spatial domains and markers. To illustrate this, we focused on two cases, one is the E7.5 mouse embryo, where the domains are roughly linearly distributed and regulators are largely known, and the other is the olfactory bulb, where the domains are not linear and the regulators are less well known. We first unsupervisedly dissected putative domains from the spatial reconstructions (gained by various methods) using *k*‐means clustering (based on the Euclidean distances of cells/samples in the embedding space). Then we match them to the positions in the original structures. Consistent with the best reconstruction by D‐CE when directly compared to domain labels (Figure 2a–c, Supporting Information), the unsupervised primary clusters (Figure 6c) and secondary clusters (Figure S16, Supporting Information) among the D‐CE reconstructed positions overlap the most with the annotated domains. From the visualization, D‐CE indeed by far best recapitulated the original domain structures and the homogeneity within domains (Figure 6c,d; and Figure S16a,b, Supporting Information), consistent with enrichment for specialized functional pathways identified among the domain specifically expressed genes (Figure 6e,f; and Figure S16c,d, Supporting Information). Such spatial domain and regionalization information unbiased revealed by de novo reconstruction will undoubtedly guide researchers to experimental explorations on the driving force and cues of pattern formation and cell migration. For the olfactory bulb data, UMAP, t‐SNE, and PCA although overall do not perform well on spatial reconstruction (Figure S17, Supporting Information), identified the spatial patterns of different layers fairly well, this might be because the cells in these different layers also happen to differ in lineages, as UMAP, PCA, and t‐SNE often separate samples by lineages (Figure 2j,k).

## 3 Conclusion and Discussion

In conclusion, we developed D‐CE which is an effective landmark free and model free de novo 3D reconstruction method for oligo and single cell analysis. Through comprehensive analysis of currently available spatial transcriptomes, we demonstrated the superior performance of D‐CE over the existing reconstruction methods on 497 reconstructions. To make the performance and advantage of D‐CE reconstruction visually apparent, D‐CE contains additional steps of specimen shape‐template fitting and marker based one‐to‐one position mapping. To evaluate the reconstruction of the 2D spatial distributions, the D‐CE 3D reconstruction results can be interpreted and evaluated by first mapping it to 2D space by template fitting and one to one mapping. When we divide the samples into different four artificial domains at midpoints of *x* and *y* coordinates, and calculate different indexes (ASI, OI, and PSImcc), these indexes confirm that of 3D model built by D‐CE after template fitting to 2D can accurately reconstruct 2D distributions of the samples (Figures 4 and 5). Furthermore, we introduce another related innovation, in comparison to previous methods in the field, D‐CE can also self‐detect spatial marker genes and use them for marker based one‐to‐one position mapping. The results, by both quantitative and visual comparisons to the original structure and to the reconstructions by other methods, revealed often magnitudes of improvements (e.g., Figures 2, 4, and 5), on average at least over fourfold improvement of D‐CE (Figure 6a,b), and visually compelling superiority of D‐CE over the existing methods. D‐CE, by accurately reconstructing the spatial patterning of the oligo and single cells, revealed many previously under or unappreciated regulators (or potential morphogens), such as oxygen, extracellular matrix, and tight junction gradients. These gradients guide the pattern formation of many biological processes, including but not limited to embryo and tissue development, regeneration, and cancer formation. Probably most fascinating, D‐CE revealed a mesoscale design principle of spatial organization which associates local network neighborhoods to spatial domains. Those interconnected mesoscale domains and their communications are fundamental in forming the microenvironment and guiding the oligo or single cells spatial distribution toward the ultimate pattern formation. The universal versatility and the accuracy of the algorithm makes it an invaluable tool for oligo or single cell analysis.

Eight of the top 10 EOC genes in mouse embryo and 8 of the top 10 EOC genes in Drosophila embryo have been reported in previous publications (Table S3, Supporting Information) to show spatial expression patterns during embryonic stage, while the one other top 10 EOC gene in mouse embryo can be verified using seqFISH data, the two other top 10 EOC genes in Drosophila embryo can be verified to have spatial expression patterns in in situ hybridization databases (Table S3 and Figure S18, Supporting Information). This reflects the thoroughness of studies on spatial transcriptions during mouse embryogenesis and the accuracy of D‐CE reconstructions. However, when the studies are less intensive, such as for the human embryonic cerebral cortex, many D‐CE self‐nominated spatial marker genes have not been reported in the literature. For example, we find oxygen binding (HBA2 and HBE1) and gap junction (DBN1 and GJA4) genes and their related pathways spatially distributed (Figure S4, Supporting Information), suggesting oxygen gradient and gap junction mediated bioelectrical field across cells might play an important yet unappreciated or underappreciated role in spatial organization in the human embryonic cerebral cortex. Experimentally, although the correspondence of domains in human brain to mouse brain is unclear, the DBN1 expression in mouse brain can be seen as spatially expressed in the mouse brain by in situ hybridization (Figure S4e, Supporting Information). These further demonstrate the accuracy and novel insight of the D‐CE in de novo discovery of spatial markers.

Then, what makes D‐CE stand out and excel previous methods for single cell 3D spatial reconstruction? Common microenvironmental cues will render the cells to have similarity in gene expression in response to the common microenvironment.^[^ ^6a,d^ ^]^ Cells of the same cell type can have different expression profiles in different spatial domains, and cells of the different cell type in the same spatial domain can have shared expression profiles, the spatially correlated transcriptome profile is the feature we want to infer to not only separate the cells of same cell type in different spatial domains, but also aggregate the cells of different cell types but in the same spatial domains. D‐CE reconstructions of spatial domain of human embryonic frontal lobe and parietal lobe demonstrate when cells of the same cell type distributed in very different spatial domains, the common spatially associated transcriptome features can be used to separate the cells of same cell type in different spatial domains. De novo reconstruction methods novoSpaRC and CSOmap, and other landmark‐based reconstruction methods, such as Satija et al.’s method,^[^ ^13a^ ^]^ also rely on this general assumption. As one would expect, cell–cell similarities reflect both lineage and spatial microenvironment effects, and direct sample wise dimensionality reduction methods, such as PCA, t‐SNE, and UMAP are indeed optimized to capture the differences in lineages (Figure 2k,j). NovoSpaRC by similarity fitting to geometric reconstruction template and CSOmap by focusing on only ligand–receptor interactions mitigate some of the confounding influences by lineages. However, by network‐reconstruction and embedding, only D‐CE is able to exploit the network of transcriptomic similarity between cells and to better capture the latent transcriptomic manifold modes of the complex biosystem that generates the data. This allows topological domains to stand out as assemblies and truly captures the design principle of spatial patterning by (micro)environment. Given that cell distributions and organizations are guided by morphogen, bioelectric voltage, and mechanical force gradients,^[^ ^6a,d^ ^]^ all these diverse types of gradients represent different forms of inter‐cellular communication in a parceled soft active matter system,^[^ ^29^ ^]^ which follow network geometry dynamics comparable to diffusion and spreading processes in social networks.^[^ ^30^ ^]^ D‐CE by embedding a local similarity network, can reliably reconstruct the spatial domains and their relationships, while the existing global transcriptome profile similarity‐based methods cannot (summarized in Figure 6). Based on the ASI, D‐CE significantly separates spatial domains and outperforms other de novo reconstruction methods. In addition, relative orientations and locations between spatial domains are also better revealed by D‐CE, as shown by PSImcc, and even the orders of samples in general are better captured by D‐CE, as shown by OI. In addition, the radius in the embedding is designed to reflect the lineage hierarchy of the cells, that is cells which are similar to many others in gene expression pattern are more central in the representation. Consistently, we find the radius in all four randomly selected single cell datasets is highly and significantly anticorrelated with the number of genes expressed (detected) across different cells, which is a reliable marker for cell stemness, and has been developed into the CytoTRACE stemness score through a smoothing operation^[^ ^31^ ^]^ (Figure S19, Supporting Information).

D‐CE is a method specifically designed and tested for 3D spatial reconstruction of oligo and single cell data by network embedding, and for this reason cannot be directly used and needs specific adjustments for nonlinear topological dimension reduction of data in general. In particular, the first step of D‐CE consists in building a gene association network by means of the connectivity specificity index (CSI), which is a procedure developed under the assumption and in the context of computational systems biology networks, therefore we cannot guarantee is valid for network reconstruction of any type of data in general. In fact, D‐CE performance is far different from routine dimensionality reduction methods such as PCA, t‐SNE, and UMAP, which we have tested and compared to and showed that do not work for spatial reconstruction as D‐CE does. D‐CE is based on biologically valid assumptions that we described above. However, when the transcriptome does not respond to local environment or form spatial domain, such as a partial transcriptome biased for cell type marker genes, the escaping cancer cells or highly mobile or shuttling cells, might defy such an assumption, and thus their locations will be hard to reconstruct. For example, MERFISH is another method generating spatial transcriptomes, however due to the limited throughput, only cell type specific marker genes were profiled, thus biasing reconstructions by all 3 spatial reconstruction methods to cell type classification, therefore we did not include the MERFISH data in the overall comparison of the 3 methods although even under this circumstances, D‐CE still performs the best (Figure S20, Supporting Information).

As D‐CE is a network embedding method, its performance strongly depends on the strategy we adopt to filter away random background level low‐strength links (which increases quadratically with the number of nodes) from the original fully connected association network. A widely used strategy is to apply a rigid threshold which cuts off the links lower than a certain strength in the association network, however this strategy does not adapt the thresholding to the local neighborhood information imprinted in the association network. CSI is a strategy developed to address this issue by adapting the thresholding to the local links’ strengths (association) pattern.^[^ ^32^ ^]^ Therefore, it is important to not only remove the global background links (such as the rigid threshold procedure does) but also to determine and remove local background level links using CSI, because this will allow to cell assemblies’ connectivity to standout in the network structure. Indeed, we find that filtering PCC using CSI performs better than directly using PD network for large datasets (more than 150 nodes/samples) as indicated by gradient down‐sampling of the BDTNP dataset (Figure S7, Supporting Information) and are used as default for all reconstructions that contain more than 150 samples.

Machine learning can be model‐based or model‐free. Methods that are model‐based require an accurate knowledge of the processes behind the 3D morphogenesis and an appropriate formalization of these processes in a mathematical model whose parameters can be learned from the data. For instance, Bayesian approaches’ parameters can be learned by maximum likelihood estimation. To the best of our knowledge, there is not yet any method for single cell 3D spatial reconstruction that is model‐based because due to the complexity of the process it is difficult to write down an accurate mathematical model whose parameters can be learned from the few and heterogeneous data currently available. In this scenario, model‐based methods would offer poor results because of the missing knowledge for modeling and poor data for learning. For this reason, current approaches for single cell 3D spatial reconstruction, such as de novo reconstruction methods novoSpaRC and CSOmap, and landmark‐based reconstruction methods, such as Satija et al.’s method 2, are data‐driven. This means that they do not exploit an explicit mathematical model of the process behind the data to predict their reconstruction, but they derive an implicit (not encoded in any mathematical formula) representation of the data directly from the data according to the assumption of the latent manifold of the data. That is, such data are the geometrical representation of the mathematical relation between the data samples, and can be learned by approximating the multidimensional similarity (or dissimilarity) relation of the data samples. Based on this assumption, a solution is to approximate the manifold similarity relations by means of a network connectivity.^[^ ^18^ ^]^

Just as spatial transcriptome can provide a blueprint for spatially mapping a gene expression, and identify spatial marker genes and spatial transcription domains, a precise in silico de novo spatial reconstruction algorithm like D‐CE/D‐CE‐t can serve the same propose—provide the spatial domain organizations and markers, without the need of expensive spatial sample preparation, and can generate the spatial information for scRNA‐seq data which lacks spatial information.

Currently all methods for 3D spatial reconstruction of single cell data including novoSpaRC and CSOmap are unsupervised for a concrete reason. A constraint to build supervised approaches is to have 3D genome‐wide single cell ground‐truth datasets on which we can implement the training phase of the supervised algorithms. The “crude” reality is that at the moment we do not have such data and therefore we cannot build supervised approaches. We hope that future efforts and improvements in ground‐truth data production could help to set an initiative such as a competition in single cell 3D spatial reconstruction similar to the effort performed in the DREAM challenge.^[^ ^33^ ^]^

## 4 Experimental Section

### Datasets for Gene Set Selection

186 Geo‐seq samples with known positions in mouse embryo E6.5, E7.0, and E7.5 and 69 scRNA‐seq datasets (GSE120963) in E7.0 mouse embryo with A and P spatial labels were used to develop the 3D reconstruction method. For better comparison with novoSpaRC, the same expression matrix as novoSpaRC is used, which is downloaded from https://www.github.com/rajewsky‐lab/novoSpaRC.^[^ ^10^ ^]^

Genes for cell–cell network construction were selected based on 3 gene lists: 4512 developmental genes based on GO database,^[^ ^34^ ^]^ which are genes with GO terms containing keywords of “differentiation,” “development,” and “morphogenesis,” 2302 transcription factors obtained from the AnimalTFs^[^ ^35^ ^]^ and RIKEN databases,^[^ ^36^ ^]^ and 4895 signaling genes obtained from the previous curation.^[^ ^37^ ^]^ All samples are first subjected to batch effect correction by ComBat. Then the batch effect corrected RPKMs (Reads Per Kilobase of exon model per Million mapped reads) are used for further analysis. All expressed genes are defined as RPKM>1 in at least 2 samples. Among them, there are 3795 developmental genes, 1646 transcription factors, and 3470 signaling genes for downstream analysis. The union, intersection, and difference of each pair of datasets or among the 3 datasets, all expressed genes and all expressed genes minus the developmental genes were used to generate a total of 19 gene lists for spatial reconstruction, gene expression levels are transformed by log _10_(FPKM + 1). For each dataset, 12 different normalization methods were applied to each gene set (Table S1, Supporting Information), which give rise to a final of 228 datasets for network construction using D‐CE. In order to embed the scRNA‐seq and Geo‐seq data together, ComBat was first used to eliminate batch effects.

The top PC loading genes are selected using function “dimdesc” R package “FactoMineR,”^[^ ^38^ ^]^ the genes with *p* value <10^−10^ are selected as top PC loading genes which result in 5731, 898, and 585 genes for PC1, 2, and 3, respectively. These 3 gene sets, individually and combined, were compared to DST genes on the performance of D‐CE. DST genes were also compared to Scialdone et al's pseudospace genes,^[^ ^7^ ^]^ which is a set of genes displaying a gradient along pseudospace axis, 334 assigned to anterior and 87 to posterior, the union of these genes are used for spatial reconstruction.

### De Novo Coalescence Embedding

A new algorithm was proposed that was named D‐CE and designed under the framework of CE methodology,^[^ ^15^ ^]^ according to which the network nodes in the embedded space were ordered preserving hidden relations of: i) homophily (similarity) on the angular coordinates and ii) hierarchy on the radial coordinates.^[^ ^15^ ^]^ In this study, the main hypothesis is that, according to CE rationale, the embedding of a developmental network of transcriptomic topological similarity between cells (an association network derived from their gene expression) should produce an angular coalescent cell ordering that recapitulates the original single cell samples’ 3D spatial tissue distribution. While the cell hierarchy on the radial coordinates is obtained via a measure of node centrality in the network topology. The details of how to implement angular and radial inference is in the network embedding sub‐section below. Indeed, any CE algorithm such as D‐CE consists of two steps (see Figure 1b): 1) network construction; 2) network embedding. In the next two sub‐sections, the specific design of each of these two steps for the proposed D‐CE is described, respectively. The Matlab code of D‐CE for de novo 3D reconstruction is an open access tool downloadable at https://github.com/JackieHanLab/D‐CE.

Step 1: Network Construction

In this section, how to build a weighted association network between Geo‐seq or cell samples in order to perform the first step of D‐CE, is described. The final weighted association network is represented as a distance adjacency matrix that is obtained from the conversion of node similarities in node distances. This association network is used in step 2 in order to perform the network embedding which provides the angular coordinates that allow the 3D spatial reconstruction. Two different strategies that can be used to build the distance matrix are proposed.

The first strategy is the following. For each normalized gene set (normalization is first done within the gene set) the pairwise distance matrix between samples is generated by using Spearman distance:^[^ ^22^ ^]^

(1) SD=1−RCC

where RCC is the Spearman correlation coefficient, or Pearson distance (PD):

(2) PD=1−PCC

where PCC is the Pearson correlation coefficient, or directly the Euclidean distance (ED) between each pair of samples.

In addition, a second strategy that is designed to apply a soft‐threshold that penalizes nontopological‐specific low correlations and rewards local connectivity similarities that are associated to high correlations, is also considered. This second strategy can be applied only to adjust correlation networks, hence it will be applied only to RCC and PCC. The first step is based on computing the Connectivity Specificity Index (CSI).^[^ ^23^ ^]^ For instance, in the case of the Pearson correlation PCC, CSI sparsifies (removing negligible links that are put to zero) the PCC similarity network according to this formula

(3) $$\begin{array}{ccc} & & \mathrm{PCC}\_{\mathrm{CSI}}_{i,j}\\ & & =\frac{\mathrm{number}\;\mathrm{of}\;\mathrm{nodes}\;\mathrm{connected}\;\mathrm{to}\;i\;\mathrm{and}\;j\;\mathrm{with}\;\mathrm{PCC}<{\mathrm{PCC}}_{i,j}-0.05}{\mathrm{number}\;\mathrm{of}\;\mathrm{nodes}\;\mathrm{in}\;\mathrm{the}\;\mathrm{network}}\; \end{array}$$

where *i* and *j* are two samples (nodes in the correlation network). The same formula can be used to compute $\mathrm{RCC}\_{\mathrm{CSI}}_{i,j}$.

The result of this first step is a similarity matrix where a zero element indicates that the similarity between two samples is negligible according to CSI. Then, the nonzero elements (*x*_+_) of this similarity matrix are “reversed” to obtain a distance matrix according to this reverse function

(4) $$f\left(x_{+}\right)\;=\;\mathrm{abs}\left(x_{+}-\min\left(x_{+}\right)-\max\left(x_{+}\right)\right)$$

where abs is the absolute value and min and max are, respectively, the minimum and maximum. Finally, after this distance matrix is created, in order to assign a distance also to nonadjacent node pairs (which are the zero elements), the shortest path between each pair of nonadjacent nodes is computed and its value is stored as their distance. This generates a PCC‐CSI distance matrix. Applying the same strategy, also the RCC‐CSI can be generated by substituting PCC with RCC in the procedure above.

In summary, 5 different distance matrices are prposed which represent 5 different network construction options for D‐CE: Pearson distance (PD), Spearman distance,^[^ ^22^ ^]^ Euclidean distance (ED), PCC‐CSI distance, and RCC‐CSI distance.

*Step 2: Network Embedding*

After getting the sample–sample distance matrix (network) according to one of the 5 different options described above, the network embedding step of the D‐CE algorithm consists of two routines.

(2.1) The first routine is associated with inferring the 3D angular coordinates of the samples and consists of two subroutines. The first subroutine is the *n*‐by‐*n* distance matrix doubly‐centering operation given by the formula

(5) $$\bar{X}=\;X-\frac{1}{n}\cdot O\cdot X-\frac{1}{n}\cdot X\cdot O-\frac{1}{n^{2}}\cdot O\cdot X\cdot O$$

where O is an *n*‐by‐*n* matrix of all 1's.

Kernel‐based machines provide a framework to generalize linear pattern recognition methods to the nonlinear domain.^[^ ^39^ ^]^ They rely on the concept of kernel trick, initially introduced by Aizerman et al.^[^ ^40^ ^]^ As a matter of fact, D‐CE (similar to any coalescent embedding algorithm^[^ ^15^ ^]^) is a nonlinear kernel‐based machine, in D‐CE the inference of the kernel is network‐topology‐driven because the distances between the points are inferred by means of the CSI filtered association network, where CSI is tailored for biological networks such as the ones here considered.^[^ ^41^ ^]^ This implies that D‐CE is able to perform nonlinear network‐driven dimension reduction, which makes this approach very different from any linear technique such as PCA. In standard kernel‐based machines, data should be centered in the feature space, by shifting the origin to the centroid of the data. Hence, D‐CE distance matrix (kernel) is also centered. From an algorithmic point of view, centering is performed easily with matrix algebra, by a subsequent column and row centering of the kernel matrix,^[^ ^15^ ^]^ according to the formula above.

However, recent studies are also advocating the option to not center the kernel,^[^ ^39^ ^]^ that is a solution that is not considered in this study because it is still under debate^[^ ^39^ ^]^ and in the previous study on coalescent embedding did not provide any major increment in performance.^[^ ^15^ ^]^

The second subroutine is the spectral decomposition of the doubly‐centered distance matrix by means of the SVD

(6) $$\bar{X}=\;U\cdot S\cdot V^{\prime }$$

(7) $$D_{n,3}=\left(\mathrm{sqrt}\left(S_{3,3}\right)\cdot \;{\left(V_{n,3}\right)}^{{}^{\prime }}\right){\;}^{\prime }$$

where *S* is an *n*‐by‐*n* diagonal matrix with singular values of $\bar{X}$ on its diagonal and sqrt is the square root operation. *U* and *V* are two unitary matrices, the columns of which are singular vectors of $\bar{X}$. *V*' is the Hermitian transpose (the complex conjugate of the transpose) of *V*. Since $\bar{X}$ is symmetric in this particular care *U* = *V*. *D*_*n*,3_ is the score matrix where each row is a node of the network and each column is a different dimension of embedding that can be used to assign to each network node respectively the *x*, *y*, *z* coordinate of the 3D embedding. Then, the coordinates are transformed into polar coordinates and the angular coordinates are kept.

(2.2) The second routine is associated with inferring the radial coordinates as a function of the node strength, which is the sum of the edge similarities incident on a node. A node with high strength is very similar to many other nodes in the network,^[^ ^15^ ^]^ therefore it is high in the topological hierarchy.^[^ ^15^ ^]^ Indeed, being similar to many nodes means that many nodes consider you at the center of the connectivity structure. Hence, according to the CE methodology,^[^ ^15^ ^]^ nodes with higher strength should be located toward the center of the embedding and therefore have lower radial coordinates; whereas nodes with lower strength should be located toward the periphery of the embedding and therefore have higher radial coordinates. On the basis of this rationale, a procedure is designed to infer the radial coordinates that is described step by step below. For a certain node *i*, its similarity is defined as

(8) $$S_{i}=\sum_{j=1}^{N}s_{i,j}\;$$

where *s*_*i*,*j*_ is the similarity metric between node *i* and *j*, and *N* denotes all nodes connected to *i*. For Spearman, Pearson, and Euclidean distance network, $s_{i,j}=\;1-d_{i,j}/\max_{i,j}d_{i,j}$, where *d*_*i*,*j*_ is the distance between node *i* and *j*, and for CSI network, *s*_*i*,*j*_ = *CSI*_*i*,*j*_ .

Nodes are first sorted in descending order by strength with nodes with highest strength ranked first. Then, the radial coordinate of the *i*th node is determined by the following formula that is termed heterogeneity‐adaptive radius (HAr) and is specifically designed for D‐CE embedding in order to capture the node hierarchy and according to the rationale that is clarified below

(9) $$HAr_{i}=\;1-\frac{\beta }{\ln\left(o_{i}\right)+1}$$

*o_i_* is the ranking value of *i*th node, and the logarithm adjustment ln (*o_i_*) of the ranking value is introduced to mitigate the growth of the denominator when the strength of a node is low and, possibly, many nodes with similarly low strength are arbitrarily ordered in the high value zone of the ranking. The adjustment coefficient $\beta \;=\frac{\mathrm{RSD}}{1+\mathrm{RSD}}\;$, where $\mathrm{RSD}\;=\frac{\mathrm{std}(S)}{\mathrm{mean}(S)}\;\;$is the relative standard deviation of the strength among all nodes, is a measure of heterogeneity of the node strength distribution.

*HAr_i_* is confined to the interval]0,1[. The reversed square brackets indicate that the value 0 and 1 are, respectively, the inferior and superior limits of the interval, but in practice they cannot be reached. For a networked system with high hierarchical organization, the node strength will have large heterogeneity because the distribution of the node strength will have high RSD and, as a consequence, *β* → 1. Hence, *β* → 1 means that the network has very high hierarchical organization and, to reflect this feature in the embedding visualization, the node with highest strength (for which *o_i_* =  1) takes *r_i_* → 0 and is located more toward the center of the embedding, then all the other nodes following it will assume larger radial values in the range]0,1[. In contrast, if the network hierarchical organization is lower than the previous case, for example, it is assumed that *β*  =  1/2, then the node with the highest strength (for which *o_i_* =  1) takes *r_i_* =  1/2, and the radial coordinates space available for the representation will be squeezed to the radial interval [0.5,1[. In conclusion, the proposed formula to infer radial node coordinates in D‐CE will visually represent networked systems with very high hierarchical organization as a 3D distribution of points occupying all the radial space from the periphery to the center of the radial coordinates. Instead, in case of networked systems with very low hierarchical organization, all the nodes will be compressed and equally distributed toward the periphery of the polar coordinate representation, and occupy only a reduced and peripheral portion of the radial space.

Note that some networked systems might have a hierarchical organization that changes across the angular coordinates with a certain pattern. In this case, the 3D embedding might result in not spherical and assume other shapes, for instance an ellipsoidal shape.

The time complexity of the method is O(N^2) where N is the number of cells, because it is constrained by the SVD, as explained in the previous study on coalescent embedding.^[^ ^15^ ^]^

### One to One Position Mapping to Template

To enable template and marker gene expression information to be used, a template containing each sample positions (not labels) is first constructed, and then optimal transportation is used to get the transport matrix between samples and positions, assuming that the sample and position weight follow a uniform distribution (p and q). D‐CE coordinates are used to calculate the Euclidean distance between each pair of cells. For the cost distance, a *k*‐nearest‐neighbor graph of the Euclidean distance matrix of samples and template is first computed, and the shortest distance between samples and positions(*C_s_* and *C_t_*) are calculated by Dijkstra algorithm, and the cost matrix C is calculated by: C  = *C*_s_  · (*p* × *q*) · *C*_t_, if marker genes are provided, the cost of gene *C*_g_ is defined as the Euclidean distance divided by the maximum Euclidean distance between samples and templates, and the total cost is calculated by the weighted sum of C and *C*_g_ and the optimal transportation is done using function “sinkhorn” in python package “POT” similar to that in novoSpaRC,^[^ ^10^ ^]^ so mask and marker information could be included. Then to make sure on sample is mapped to only one position, the position‐cell pair is first selected that have maximum weight in the optimal transport matrix, and map the cell to the position, then the node‐cell pair is removed, and the same mapping is repeat until each the cell is mapped to a position in the template.

To facilitate single cell resolution mapping, as an additional option of D‐CE‐t package (“‐scmap”), after reconstruction, single cell samples can be also mapped to the spatial location with maximum probability.

### Optimizing Normalization and Edge Weight for Reconstruction

The 3D coordinates of each sample from each of the 60 possible strategies of D‐CE (12 normalization methods and 5 edge weighting distances) were obtained to evaluate the performance of each of them in spatial reconstruction as follows.

For spatial reconstruction, the anterior and posterior samples are further divided into 4 groups according the spatial locations: dA, dP, pA, and pP, which means the distal/proximal part of anterior/posterior, the four groups are colored with red, green purple and yellow, respectively, in Figure 2. For each germ layer in each stage, only the angular coordinates of the samples (with the radius all set to 1) are used to calculate the following three indexes. 1) The angular separability index (ASI) of each part from the rest of the samples is defined and implemented by Muscoloni et al.;^[^ ^15^ ^]^ 2) The projection separability index—Matthews correlation coefficient (PSImcc) is calculated by the mean value of Matthews correlation coefficient between the order of cells from each pair of clusters (groups) and the order that well separate the 2 clusters when all the cells from those 2 clusters are projected to the line that link the median of the 2 clusters. For each pair of clusters cluster 1 and cluster 2, which have *n*_1_ samples in cluster 1 and *n*_2_ samples in cluster 2, labels: L: {*l_n_* |n ∈ 1: (*n*_1_ + *n*_2_),*l_n_* ∈ {0, 1}} where 0 present cluster 1 and 1 present cluster 2, the samples are first projected to the line that link the center of the 2 clusters, then, the samples can be divided into 2 clusters according to the projected positions of the samples, label *n*_1_ samples in one side to be cluster 1’ and *n*_2_ samples in the other side to be cluster 2’ then labels L_2_: {*l_n_* |n ∈ 1: (*n*_1_ + *n*_2_),*l_n_* ∈ {0, 1}} then, the comfusion matrix is calculated and Matthews correlation coefficient is calculated by $\mathrm{mcc}\;=\;\frac{TP*TN-FP*FN}{\sqrt{\left(TP+FP)*(TP+FN)*(TN+FP)*(TN+FN\right)}}$, where TP is “True Positives,” FNis “False Negatives,” FP is “False Positives,” and TP is “True Positives,” then, since the position of the 2 clusters are unknown, a comparison of the mcc is taken when label the samples in each of the 2 side as cluster 1, and take the maximum to be PSImcc. 3) The ordering index (OI) is used to compare the order of samples to the known order of samples. The OI of A, P, L, or R samples was calculated in each reconstruction between their original proximal to distal orders and their orders in reconstructed coordinates. The original order is determined by the labels of samples, and the rank/order of samples in the reconstructed spatial structures are determined by

a) First OI calculated for the Geo‐seq samples in each spatial domain, A, P, L, or R, separately. For each domain, the reconstructed spatial positions of samples from each layer were obtained, if there are more than one sample in the same layer, the geometric center of all the samples in this layer is used as the spatial position;
b) The center of the first layer samples is designated as the reconstructed position of the first layer, then rank the sequential layers by the shortest spatial distance to the last layer.
c) The RCC of the original and reconstructed ranks are calculated as OI.
d) The minimal OI in A, P, L, and R is taken as the overall OI of the reconstruction.

For the BDTNP dataset, the embryo is cut into 4 groups according to the *x* and *z* coordinates in original locations, then the angular coordinates are used to calculate ASI and PSImcc as described above. Same as the mouse embryo, the fly embryo is bilaterally symmetric along *y* axis, therefore only half of the embryo is used as novoSpaRC did. As the order of cells on *y* axis was not considered by novoSpaRC,^[^ ^10^ ^]^ the reconstructed spatial order is also evaluated in *x* and *z* axes using the ASI and PSImcc of the 4 groups according to *x* and *z* axes. For OI, the *x*, *y*, or *z* coordinate is sorted from high to low and divided into 10 groups, the original order of other groups is determined by the ascending order of coordinate, the geometric center of each groups in the reconstructed structure is calculated as the spatial location of this group. The group with lowest mean coordinate is designated as the first group, then the rest are calculated as described above for Geo‐seq data.

### Comparing Dimensionality Reduction Methods in Spatial Reconstruction

Besides D‐CE, 5 other dimensionality reduction methods, PCA, t‐SNE, and UMAP are also tested for spatial reconstruction in Cartesian coordinates, and then they are compared with D‐CE using ASI, PSImcc, and OI. The 3 indexes of all the results using D‐CE, PCA, t‐SNE, and UMAP are ranked in descending order, and the maximum ranks are calculated as described above.

### Comparison to the Existing De Novo Spatial Reconstruction Method

For CSOmap, the TPM of all LR genes were used for spatial reconstruction. Only human and mouse LR interaction were provided.

As novoSpaRC method is developed specifically for Berkeley Drosophila Transcription Network Project (BDTNP) dataset, which only contains the expression level of 84 TFs. For comparison to novoSpaRC, the D‐CE of the BDTNP data used all 84 TFs’ profiles with the same normalization method and distance metric as for to the 3D reconstruction of the Geo‐seq data.

To determine the coordinates of samples reconstructed by novoSpaRC, the probabilistic coupling matrix $T_{+}^{m\times n}\;$between m samples and *n* locations is calculated using novoSpaRC, then the dot product $T_{+}^{m\times n}\cdot L^{n\times 3}$, where *L* ^*n* × 3^ is the original 3D coordinates of the locations, is used to determine the reconstructed sample locations. Specifically, *T*_*i*,*j*_is the probability of sample *i* mapping to location *j*, and as the distribution of $\sum_{j}T_{i,j}$ follow a uniform distribution, the weighted sum coordinates of all the locations for sample *i* $\left(\sum_{j\;=\;1}^{n}T_{i,j}\cdot x_{j},\;\sum_{j\;=\;1}^{n}T_{i,j}\cdot y_{j},\;\sum_{j\;=\;1}^{n}T_{i,j}\cdot z_{j}\right)$ is is used to as novoSpaRC reconstructed location (with *x*, *y*, and *z* coordinates) of sample *i*, where(*x_j_*,*y_j_*,*z_j_*) is the coordinate of the location *j*.

### Comparison to the Existing De Novo Spatial Reconstruction Method—Identifying Spatial Domains and Corresponding Gene Expression Signatures

Putative domains are obtained by K means clustering of the samples according to the coordinates in the reconstructed structure based on the elbow method (Figure S21, Supporting Information), then the top 500 domain specifically highly expressed genes were determined by log fold‐changes of the samples within the domain versus outside of the domain. Then GSEA on KEGG pathway is performed by the rank of the log fold‐changes.

### Down‐Sampling of the BDTNP Dataset and Comparison of PCC versus PCC‐CSI Network

The samples in the BDTNP dataset are randomly selected to 1/*n*, with *n* = 2–100, of the total number of samples. The sampling is repeated 20 times at each sampling rate. For each down‐sampled BDTNP sample set, the PD network and PCC‐CSI networks are used for D‐CE. ASI and PSImcc are calculated and the mean ASI and PSImcc of the 20 repeats at each sampling rate are plotted to compare the performance PD network and PCC‐CSI network, on samples of different sizes.

### Reconstruction of Transcriptome Data with Known Spatial Domain Labels

Log2 transformed FPKM (Fragments Per Kilobase of exon model per Million mapped fragments) was used for D‐CE and novoSpaRC. TPM (Transcripts Per Kilobase of exon model per Million mapped reads) was used for CSOmap reconstruction as required. The spatial order of the samples is reconstructed using D‐CE, novoSpaRC and when applicable CSOmap (only applicable to human and mouse LR‐including gene sets). To evaluate the reconstructed spatial gene expression patterns for the Drosophila and zebra fish embryo scRNA‐seq datasets, as there is no specific label for each sample, the coarse‐grain domains were obtained on the matching FISH image by first converting it to gray scale, and then cut into 10 layers along either anterior‐posterior or dorsal‐ventral axis. The gray‐scale density of each layer was used as the gold standard expression level in each layer. The direction of the reconstructed structure is corrected by rotating around *x* and *y* axis with different angles (with *π*/30 as step size, sampled from 0 to 2*π*) and cut into 10 layers according to the *x* and *z* axis. Then the RCC between FISH order and each rotated reconstructed structure layer order were calculated. The orientation with max RCC is defined as the optimal orientation, whose OI is used as the final OI.

### Reconstruction of Transcriptome Data with Spatial Coordinates in a Template

For 8 transcriptome data with spatial coordinates, such as barcoded microarray‐based spatially resolved transcriptome, LCM‐seq, seqFISH, and MERFISH data, the spatial order or coordinates in a template as gold standard is used to evaluate the reconstructed structure of each method. The log2 transformed gene expression values were used for reconstruction. For each experiment, samples are labeled by their *x* and *y* coordinates and used as gold standards to compute OI, ASI, and PSImcc after the embedding of the samples.

### Expression Order Correlation (EOC) Calculation and Spatial Marker Gene Selection

The reconstructed coordinates are rotated 30 times along the XY and XZ planes respectively (12 degrees each time) to calculate the RCC between the gene expression level and the rotated coordinate X, arriving at a 30 by 30 matrix of RCC. Then the 30 by 30 RCC matrix is converted into a vector of 900 values, and clustered into 2 and 3 clusters by K‐means for 2D and 3D templates, respectively. The top EOC gene in the large and small clusters are the selected as the top 1 and 2 spatial marker genes for D‐CE‐t as default. NovoSpaRC randomly selected 1 or 2 markers for marker‐based template fitting, but it was done 100 times to select the best one for a favorable result for novoSpaRC.

### GSEA Analysis for EOC Genes

Top 5% EOC genes in each dataset were used for GO and KEGG GSEA analysis with genes’ EOC as the rank and genesets with each GO and KEGG term as signatures. The function “GSEA” in R package “clusterProfiler” was used with “pvalueCutoff = 0.05, pAdjustMethod = fdr, maxGSSize = 500 (if the gene number is more than 500, than maxGSSize = 1000)”, and default settings for other parameters.

### Whole Mount In Situ Hybridization Experiment

Mid‐gastrulaton stage (embryonic day 7.5, E7.5) embryos were collected from C57BL/6 mice, and embryos images were taken for recording and confirmation of developmental staging. Animal procedures conducted in this study were approved by the Institutional Animal Care and Use Committee of Guangzhou Institutes of Biomedicine and Health, Chinese Academy of Sciences (Institutional Animal Welfare Assurance Number N2022102).

## Conflict of Interest

The authors declare no conflict of interest.

## Author Contributions

Y.Z. and S.Z. contributed equally to this work. J.D.J.H. conceived the project. J.D.J.H. designed, with C.V.C.’s help, the project and analyses. J.D.J.H. and C.V.C invented the network construction, J.D.J.H. and Y.Z. invented de novo marker identification and one‐to‐one template fitting part of D‐CE. C.V.C. invented the network embedding part of D‐CE and designed the algorithm. Y.Z. and S.Z. wrote, verified, and tested the codes with occasional help from C.V.C. J.D.J.H., C.V.C., Y.Z., and S.Z. wrote the paper. J.X. did in situ hybridization supervised by G.P., Y.Y. prepared embyos.

## Supporting information

Supporting Information

## Data Availability

All data analyzed are openly available in public repositories with accession numbers indicated in the Experimental Procedures.
