# Supplementary material for: Spatial Reconstruction of Oligo and Single Cells by De Novo Coalescent Embedding of Transcriptomic Networks
Source: Adv Sci (Weinh). 2023 Jun 15;10(20):2206307. doi: 10.1002/advs.202206307 (PMC10369275; doi:10.1002/advs.202206307)
Supplement: Supplementary file 1 — Supporting Information [file ADVS-10-2206307-s001.pdf]

ADVANCED SCIENCE
Open Access

# Supporting Information

for *Adv. Sci.*, DOI 10.1002/advs.202206307

Spatial Reconstruction of Oligo and Single Cells by De Novo Coalescent Embedding of Transcriptomic Networks

*Yuxuan Zhao, Shiqiang Zhang, Jian Xu, Yangyang Yu, Guangdun Peng, Carlo Vittorio Cannistraci* and Jing-Dong J. Han**

Supporting Information

**Spatial Reconstruction of Oligo and Single Cells by De Novo Coalescent Embedding of Transcriptomic Networks**

*Yuxuan Zhao#, Shiqiang Zhang#, Jian Xu, Yangyang Yu, Guangdun Peng*

*Carlo Vittorio Cannistraci* and Jing-Dong J. Han**

Suppl. Algorithmic procedures for D-CE and D-CE-t, and for OI, PSI-mcc, ASI and EOC calculations

D-CE algorithm:

INPUT: $x_{n,g}$ (expression matrix of n samples and g genes)

OUTPUT: $D_{n,xyz}$(the cartesian coordinate of each sample)

(1) Network construction

A normalized expression matrix $xn_{n,g}$ in which each element is the square root of each element in the $x_{n,g}$ is first calculated, and a PCC network $P_{n,n}$ is built as follow:

For i=1...n

For j=1...n

$P_{i,j}$ is the Pearson correlation coefficient of row i and j

$$P_{i,j}=\frac{E(xn_{i,}\cdot xn_{j,})-E(xn_{i,})E(xn_{j,})}{\sqrt{E(xn_{i,}^2)-E(xn_{i,})^2}\sqrt{E(xn_{j,}^2)-E(xn_{j,})^2}}$$

Then, the distance adjacency network $dist_{n,n}$ is calculated:

if CSI matrix is used

CSI matrix is calculated as:

for i=1..n

for j=1...n

$$CSI_{i,j} = \frac{sum(P_{i,} < (P_{i,j} - 0.05) \& P_{,j} < (P_{i,j} - 0.05)}{number\ of\ nodes\ in\ the\ network}$$

The nonzero elements ($CSI_+$) of this similarity matrix are ‘reversed’ to obtain a distance matrix:

for i=1...n

for j=1...n

if $CSI_{i,j}$=0

$dist_{i,j}$=0

else

$dist_{i,j}$=|$CSI_{i,j} - \max(CSI_+) - \min(CSI_+)$|

The distance between nonadjacent nodes is set as the shortest path:

for i=1..n

for j=1...n

If $CSI_{i,j}$==0

$dist_{i,j}$=shortest path between node i and j

else

Just use Pearson distance as the weight directly:

$dist_{n,n} = 1 - P_{n,n}$

[43] Network embedding

(2.1) Then, centered distance matrix $\overline{dist_{n,n}}$ is built:

$$\overline{dist_{n,n}} = dist_{n,n} - \frac{1}{n} \cdot O \cdot dist_{n,n} - \frac{1}{n} \cdot dist_{n,n} \cdot O - \frac{1}{n^2} \cdot O \cdot dist_{n,n} \cdot O$$

where $O$ is an n-by-n matrix of all 1's;

Then, apply SVD on the centered distance matrix and get the 3D coordinate:

$\bar{X} = U \cdot S \cdot V'$

$D_{n,xyz} = (sqrt(S_{3,3}) \cdot (V_{n,3})' )'$

(2.2) Radial coordinate adjustment:

if CSI matrix is used

for i = 1...n

$$S_i = \sum_{j=1}^{N} CSI_{i,j}$$

else

for i = 1...n

$$S_i = \sum_{j=1}^{N} (1 - dist_{i,j} / \max_{i,j} dist_{n,n})$$

$$RSD = \frac{std(S_n)}{mean(S_n)}$$

$$\beta = \frac{RSD}{1 + RSD}$$

The final Cartesian coordinate $D_{n,xyz}$ is calculated:

Sort the nodes according to $S_n$ in descending order to ranks $r_{1\dots n}$;

for i = 1...n

for j in x, y, z

Compute the original radius $R_i^{svd} = \sqrt{(D_{i,x})^2 + (D_{i,y})^2 + (D_{i,z})^2}$

Compute the final Cartesian coordinates:

$$\widetilde{D}_{i,j} = \frac{D_{i,j}}{R_i^{svd}} * (1 - \frac{\beta}{\ln(r_i)+1})$$

D-CE-t algorithm:

INPUT: $KS_{n,n}$, $KT_{n,n}$, $C_g, P_{n,d}$ (knn graph of samples and positions on the template, and the cost (Euclidean distance divided by maximum of the distance between the D-CE coordinates and the template coordinates) between samples and positions and spatial coordinates of n positions in d dimension)

OUTPUT: $D_{n,d}$(the cartesian coordinate of each sample)

the distance of samples and positions on the knn graph are used as the cost:

$C_s = Dijkstra(KS_{n,n})$

$C_t = Dijkstra(KT_{n,n})$

The cost between samples and positions: $C = C_s \cdot (p \times q) \cdot C_t$

Option:

If $C_g$ is given:

$C = a \cdot C + (1 - a) \cdot C_g$

$OT_{n,n} = \text{sinkhorn}(p, q, C)$

Then one to one position mapping matrix $D_{n,d}$ is built as follow:

$N_1$: set of positions that haven't been mapped to the node: $N_1 = 1{:}n$

$n_1$: number of positions that haven't mapped to the node: $n_1 = n$

While $n_1 > 0$

If $OT_{i,j} = \max(OT_{N_1,N_1})$

$D_{i,} = P_{j,}$

$n_1 = n_1 - 1$

$N_1 = N_1 - \{i\}$

ASI calculation:

INPUT: $\theta_{1\ldots n}, \varphi_{1\ldots n}$ $L_n$(the angular coordinate and the spatial location (dA, dP, pA, pP) of each sample)

OUTPUT: ASI

For each spatial location $l$:

$\varphi_{ext1}^{l} = \min_{i=1\ldots N^l} \varphi_i^l$

$\varphi_{ext2}^{l} = \max_{i=1\ldots N^l} \varphi_i^l$

let $r_{1\ldots N^l}^{l}$ be a subset of rank of samples $r_{1\ldots n}$ according to $\theta$

sort the rank $r_{1\ldots N^l}^{l}$ obtaining $s_{1\ldots N^l}^{l}$

for i in 1…$N^l - 1$

w(i)= $s_{i+1}^l - s_i^l - 1$

w($N^l$)= $N - s_{N^l}^l + s_1^l - 1$

Let $\theta_{ext1}^l, \theta_{ext2}^l$ be the 2 extremes, k is the index to maximize w(i)

If k=$N^l$

Select the samples that $\varphi_{ext1}^l < \varphi < \varphi_{ext2}^l$ and $\theta_{ext1}^l < \theta < \theta_{ext2}^l$

| |
|---|
| These samples will be mapped to 2D space using:<br>$$y = \varphi - \varphi^{l}_{ext1}$$<br>$$x = \theta - \theta^{l}_{ext1}$$<br>Else<br>Select the samples that $\varphi^{l}_{ext1} < \varphi < \varphi^{l}_{ext2}$ and $\theta^{l}_{ext1} > \theta$ or $\theta < \theta^{l}_{ext2}$<br>These samples will be mapped to 2D space using:<br>$$y = \varphi - \varphi^{l}_{ext1}$$<br>$$x = mod(\theta + 2 * \pi - \theta^{l}_{ext2}, 2 * \pi)$$<br>then, a convex hull of samples in location l is calculated, and $w^{l}$is defined as the number of the samples that fall into the convex hull but not in location l<br>The overall ASI is calculated by<br>$$\mathrm{ASI} = 1 - \frac{\mathrm{sum}_{l}\, w^{l}}{\mathrm{sum}_{l}\, w^{l}_{worst}}$$<br>Where $w^{l}_{worst} = ceil((N - N^{l}) * \frac{N^{l}-1}{N^{l}})$ |
| OI calculation:<br>INPUT: $C_{n,xyz}$, $D_n$, $L_n$ (the cartesian coordinate, the spatial domain each sample belonged to and the known spatial layer each sample belonged to)<br>OUTPUT: OI (order index)<br><br>For each domain d in $D_n$<br>$\mathrm{C1}_{nd,xyz} = C_{D_n==d,}$<br>$\mathrm{L1}_{nd} = L_{D_n==d}$<br>for i = 1... $(\max_{nd} L1 - \min_{\mathrm{nd}} L1 + 1)$<br>$C2_{n^{i},3} = C1_{\mathrm{L1}==(\min_{nd}\mathrm{L1}-1+i),}$<br>$Cen_i = (\frac{\sum_{j=1}^{n^{i}} C2_{j,x}}{n^{i}}, \frac{\sum_{j=1}^{n^{i}} C2_{j,y}}{n^{i}}, \frac{\sum_{j=1}^{n^{i}} C2_{j,z}}{n^{i}})$<br>The rank of reconstructed spatial order $Rr$ is calculated as:<br>for i = 1 ... $(\max_{n^d} L1 - \min_{n^d} \mathrm{L1} + 1)$<br>$Rr_i = 0$ |

$Rr_1 = 1$

While min($r^R$)=0

$$\text{Cmax} = Cen_{Rr==\max(Rr)}$$

for each i that $Rr_i = 0$

$$\mathrm{d}_i = \sqrt{(Cen_{i_x} - \text{Cmax}_x)^2 + (Cen_{i_y} - \text{Cmax}_y)^2 + (Cen_{i_z} - \text{Cmax}_z)^2}$$

$$\text{Rr}_{\mathrm{d}_i == \min_{Rr_i=0} \mathrm{d}_i} = \max(Rr) + 1$$

The rank of original spatial order $Ro = 1 \ldots (\max_{nd} L1 - \min_{\text{nd}} L1 + 1)$

The OI is defined as the rank correlation of $Rr$ and $Ro$

$$\text{OI}_a = \frac{E(Rr \cdot Ro) - E(Rr)E(Ro)}{\sqrt{E(Rr^2) - E(Rr)^2}\sqrt{E(Ro^2) - E(Ro)^2}}$$

Finally, the overall OI is defined as the minimal OI in all of the domains

$$OI_{final} = \min_d OI_d$$

PSImcc Calculation:

INPUT: $D_{n,xyz}$, $L_n$(the Cartesian coordinate and the spatial location (dA, dP, pA, pP) of each sample)

OUTPUT: PSImcc (Projection separability index- Matthews correlation coefficient)

First, the Cartesian coordinates of the geometric center of each spatial part is calculated:

for i in dA, dP, pA, pP

$$\text{D1}_{ni,xyz} = D_{L_n==i,}$$

$$M_i = (median(\text{D1}_{,x}), median(\text{D1}_{,y}), median(\text{D1}_{,z}))$$

For i, j in each pair of spatial part, samples in these parts are projected onto the line linking the two median coordinates:

Denote the Cartesian coordinate of the sample as S,

$$P_{Ln==i \text{ or } Ln==j} = M_i + \frac{(\text{S} - M_i) \cdot (M_j - M_i)}{(M_j - M_i) \cdot (M_j - M_i)} * (M_j - M_i)$$

Then, the samples in one side are considered as spatial part $i$ while other side is part $j$, we considered each part as part $i$ and calculate MCC between

original spatial label and projected order as:

$$\text{mcc} = \frac{TP*TN-FP*FN}{\sqrt{(TP+FP)*(TP+FN)*(TN+FP)*(TN+FN)}}$$

and take the maximum of the 2 mcc as the mcc of the 2 groups:

$$mcc_{ij} = \max(mcc_1, mcc_2)$$

The PSImcc value is defined as:

$$\text{PSImcc} = \text{mean}(mcc_{ij})/(1 + \text{std}(mcc_{ij}))$$

EOC calculation:

INPUT: $O_n$ (observed order of n samples), $P_{n,3}$(spatial coordinates of n samples)

OUTPUT: EOC

For i =1,2…30

For j =1,2…30

$P^*_{n,3}$ are the coordinates of samples after being rotated with an azimuth angle $2 * \frac{\pi}{30} * \text{i}$ and a polar angle$2 * \frac{\pi}{30} * \text{j}$

Cor[i,j] is the spearman correlation between $P^*_{,1}$ and $O_n$

$\text{EOC} = \max_{i,j} Cor$

## Supplementary Tables

**Supplementary Table 1. 12 normalization methods evaluated for spatial reconstruction**

| Method | Description |
|---|---|
| None | without normalization |
| DRS | dividing by the sum within rows (cells) |
| DCS | dividing by the sum within columns (genes) |

| | |
|---|---|
| LOG | logarithm with base 10 of each matrix element plus 1 |
| ZSCORE | ZSCORE within columns |
| ZSCORET | ZSCORE within rows |
| QUANTILE | quantile normalization within columns |
| QUANTILE T | quantile normalization within rows |
| SQRT | square root of each element of the matrix |
| MANORM | dividing by the column mean |
| PARETO SCALING | each column is centered to have mean of 0 by subtracting the column mean from each value in the column |
| PLUS(ABS(MIN)) | adding to each matrix's element the minimum value in the matrix, in absolute value. |

**Supplementary Table 2. Spatially labeled transcriptome datasets tested for spatial reconstruction and number of reconstructions per dataset**

| **Dataset** | **Number of reconstructions** | **Single cell**<br><br>**Yes/No** | **Ground truth available** | **Data access** |
|---|---|---|---|---|
| Mouse embryo Geo-seq | 10 | No | Yes (domain) | GSE120963 |
| Human cerebral cortex frontal lobe scRNA-seq | 1 | Yes | Yes (domain) | GSE103723 |
| Human cerebral cortex parietal lobe | 1 | Yes | Yes | GSE103723 |

| | | | | |
|---|---|---|---|---|
| scRNA-seq | | | (domain) | |
| Mouse neocortical layer microsurgical RNA-seq | 1 | No | Yes (domain) | GSE27243 |
| Mouse embryo brain ISH | 1 | Single cell resolution | Yes (template) | S-EPMC4381528 |
| BDTNP | 1 | Single cell resolution | Yes (template) | GSE113576 |
| Mouse olfactory bulb | 12 (arrays) | No | Yes (template) | www.spatialtranscriptomicsresearch.com |
| Human breast cancer | 4 (arrays) | No | Yes (template) | www.spatialtranscriptomicsresearch.com |
| Cancerous prostate | 12 (arrays) | No | Yes (template) | www.spatialtranscriptomicsresearch.com |
| Melanoma lymph node | 8 (arrays) | No | Yes (template) | www.spatialtranscriptomicsresearch.com |
| Postmortem lumbar and cervical spinal cord tissue | 407 (arrays) | No | Yes (template) | https://als-st.nygenome.org |
| Mouse hippocampus | 21 (arrays) | Single cell resolution | Yes (template) | http://dx.doi.org/10.1016/j.neuron.2016.10.001. |
| Mouse brain | 14 (arrays) | Single | Yes | GSE98674 |

| | | | | |
|---|---|---|---|---|
| | | cell resolution | (template) | |
| Mouse medial ganglionic eminence | 4 (arrays) | No | Yes (template) | GSE60402 |
| Total | 497 | 6 / 14 | 14 / 14 | |

**Supplementary Table 3. Top 10 EOC genes in Drosophila and mouse embryo datasets and the evidence in published papers or in situ databases to validate the expression patterns**

| Species | Gene | Paper/Database | PMID/link |
|---|---|---|---|
| Drosophila | Ilp4 | Progress in the characterization of insulin-like peptides in aphids: Immunohistochemical mapping of ILP4 | 34246764 |
| Drosophila | CG11208 | Berkeley Drosophila Genome Project (fruitfly.org) | https://insitu.fruitfly.org/cgi-bin/ex/report.pl?ftype=10&ftext=FBgn0034488 |
| Drosophila | CenG1A | Downregulation of Centaurin gamma1A increases synaptic transmission at Drosophila larval neuromuscular junctions | 25074496 |
| Drosophila | gk | Identification of the glycerol kinase gene and its role in diapause embryo restart and early embryo development of | 24365596 |

| | | | |
|---|---|---|---|
| | | Artemia sinica | |
| Drosophila | Mes2 | Mes2, a MADF-containing transcription factor essential for Drosophila development | 17029287 |
| Drosophila | Dfd | The spatial and temporal deployment of Dfd and Scr transcripts throughout development of Drosophila | 2450726 |
| Drosophila | ImpE2 | The Drosophila IMP-E2 gene encodes an apically secreted protein expressed during imaginal disc morphogenesis | 2115480 |
| Drosophila | sna | Interacting functions of snail, twist and huckebein during the early development of germ layers in Drosophila | 8026325 |
| Drosophila | tsh | Zinc-finger paralogues tsh and tio are functionally equivalent during imaginal development in Drosophila and maintain their expression levels through auto- and cross-negative feedback loops | 19097089 |
| Drosophila | bmm | Berkeley Drosophila Genome Project (fruitfly.org) | https://insitu.fruitfly.org/cgi-bin/ex/report.pl?ftype=10&ftext=FBgn0036449 |
| Mouse | Cdx2 | Expression of Cdx-2 in the | 8573715 |

| | | | |
|---|---|---|---|
| | | mouse embryo and placenta: possible role in patterning of the extra-embryonic membranes | |
| Mouse | Hoxb1 | Acquisition of Hox codes during gastrulation and axial elongation in the mouse embryo | 12835396 |
| Mouse | Cdx1 | Cdx1::Cre allele for gene analysis in the extraembryonic ectoderm and the three germ layers of mice at mid-gastrulation | 19241391 |
| Mouse | Hoxa1 | An evolutionary conserved element is essential for somite and adjacent mesenchymal expression of the Hoxa1 gene | 9438427 |
| Mouse | Cxx1c | Selective expression of sense and antisense transcripts of the sushi-ichi-related retrotransposon – derived family during mouse placentogenesis | 25888968 |
| Mouse | T | The T-box transcription factor Eomesodermin is essential for AVE induction in the mouse embryo | 23651855 |
| Mouse | Hes7 | https://crukci.shinyapps.io/Spati | |

| | | | |
|---|---|---|---|
| | | alMouseAtlas/ | |
| Mouse | Aldh1a2 | Retinoic acid is involved in the metamorphosis of the anal fin into an intromittent organ, the gonopodium, in the green swordtail (Xiphophorus hellerii) | 24204880 |
| Mouse | Ifitm1 | In vivo functional requirement of the mouse Ifitm1 gene for germ cell development, interferon mediated immune response and somitogenesis | 23115618 |
| Mouse | Hoxb2 | Patterning in time and space: HoxB cluster gene expression in the developing chick embryo | 25602523 |
| Mouse | Tbx6 | Critical role for Tbx6 in mesoderm specification in the mouse embryo | 12915233 |
| Mouse | Msgn1 | Interaction of Wnt3a, Msgn1 and Tbx6 in neural versus paraxial mesoderm lineage commitment and paraxial mesoderm differentiation in the mouse embryo | 22546692 |

## Supplementary Figures

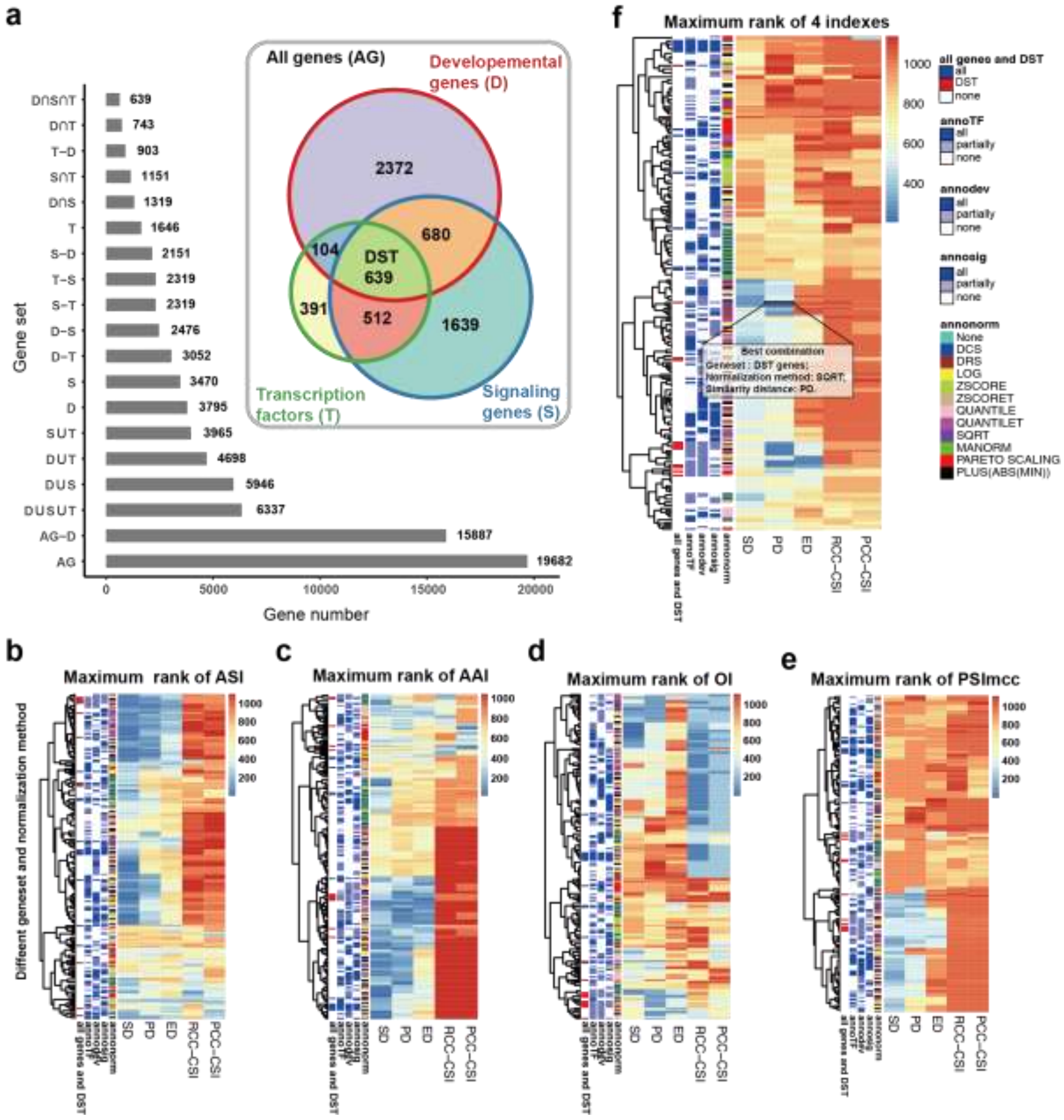

**Supplementary Figure 1. D-CE parameter optimization using Geo-seq data. a,** Various combinations of 3 different gene sets tested for Geo-seq data spatial reconstruction. These include 3795 developmental genes, 3470 signaling genes and 1646 transcription factors. Then the union, intersection and each unique part from these 3 gene sets were derived into a total of 19 gene meta-sets from the non-overlapping 7 sections in the Venn diagram. We tested all 19 gene sets for their

performance in retrieving the spatial information of the samples. **b to e,** The maximum rank of ASI, OI and PSImcc in Geo-seq dataset with 19 gene sets and 12 normalization methods ('annonorm'). Here, we compared 5 distance matrices, including Spearman distance [22], Pearson distance (PD), Euclidean distance (ED) and CSI filtering on PCC and RCC (PCC-CSI and RCC-CSI). For the 19 gene sets, we annotated them with 'annoTF', 'annodev', and 'annosig' to show whether one gene set contain or partially contain genes from TFs ('annoTF'), developmental related genes ('annodev') and signaling ('annosig') genes. All genes and DST genes were specifically annotated with 'all gens and DST'. **f**, The maximum rank of the 4 indexes in Geo-seq dataset for spatial reconstruction.

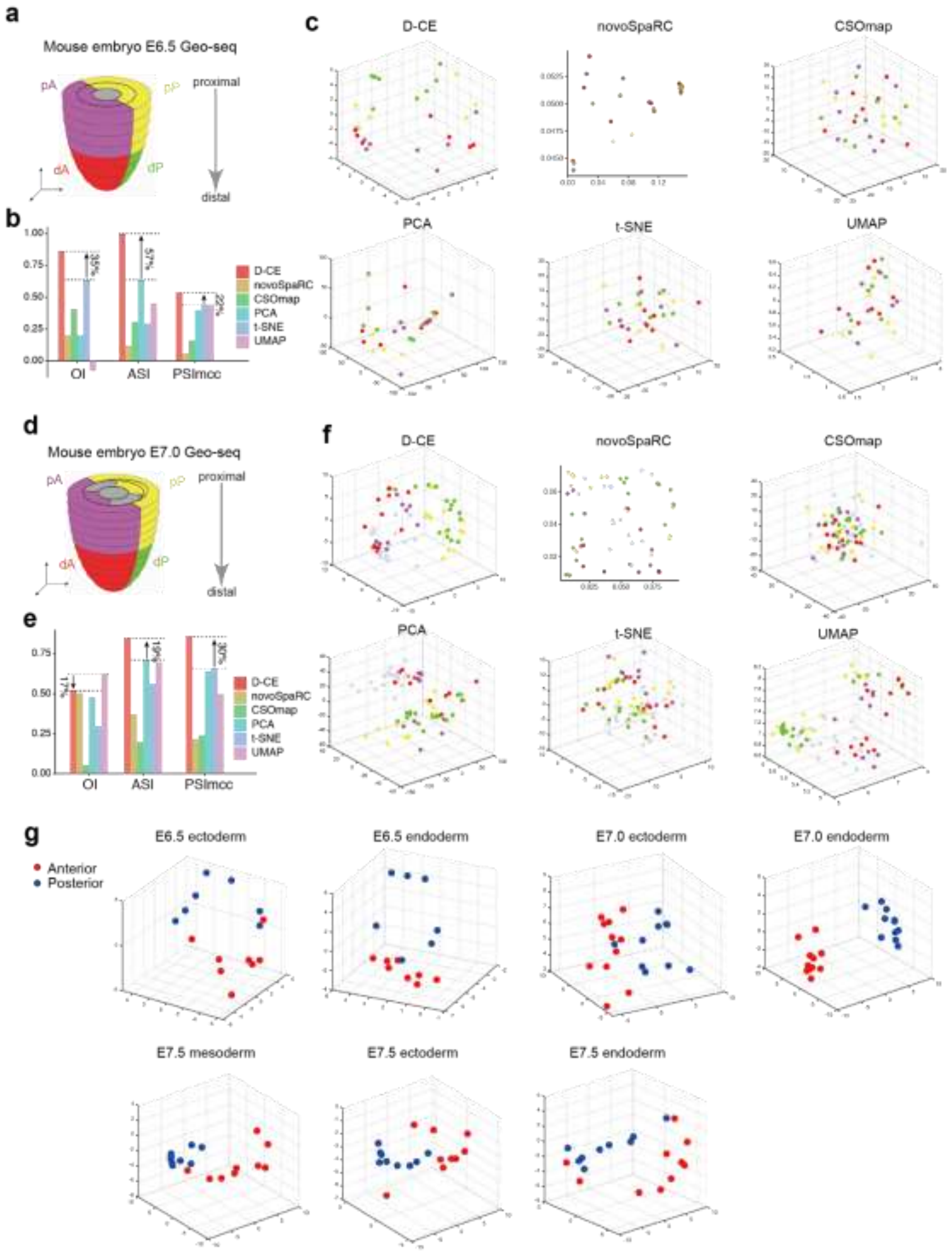


**Supplementary Figure 2. Reconstruction of spatial domain labels mouse embryo E6.5, E7.0 and E7.5 data by D-CE, novoSpaRC, CSOmap, PCA, t-SNE and UMAP. a to f,** The same layout as Figure 2a to c but for E6.5 (a to c) and E7.0 (d to f) with all germ layers combined together. **g**, D-CE spatical reconstruction results of mouse E6.5, E7.0 and E7.5 Geo-seq data with each germ layer separately, and colored by anterior [44] or posterior (blue).

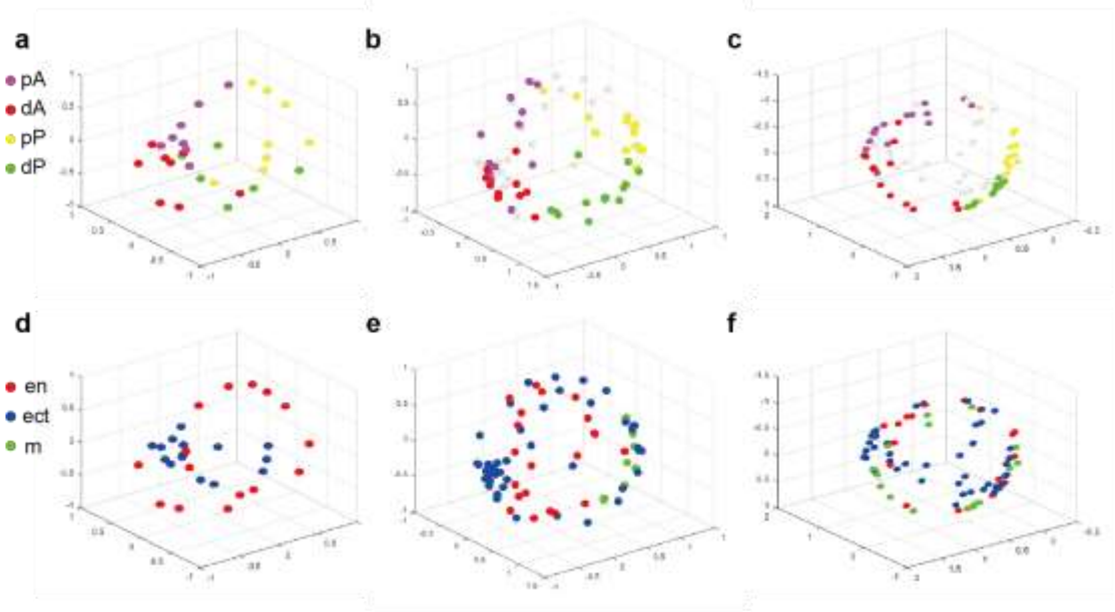


**Supplementary Figure 3.** The spatial distribution of different germ layers, **a**, **b**, **c**, Spatial reconstruction of E6.5, E7.0, and E7.5 mouse embryo, colored by domain information, **d**, **e**, **f**, The same reconstructed structure as a, b and c, samples colored by endoderm [44], mesoderm (green) and ectoderm (blue).

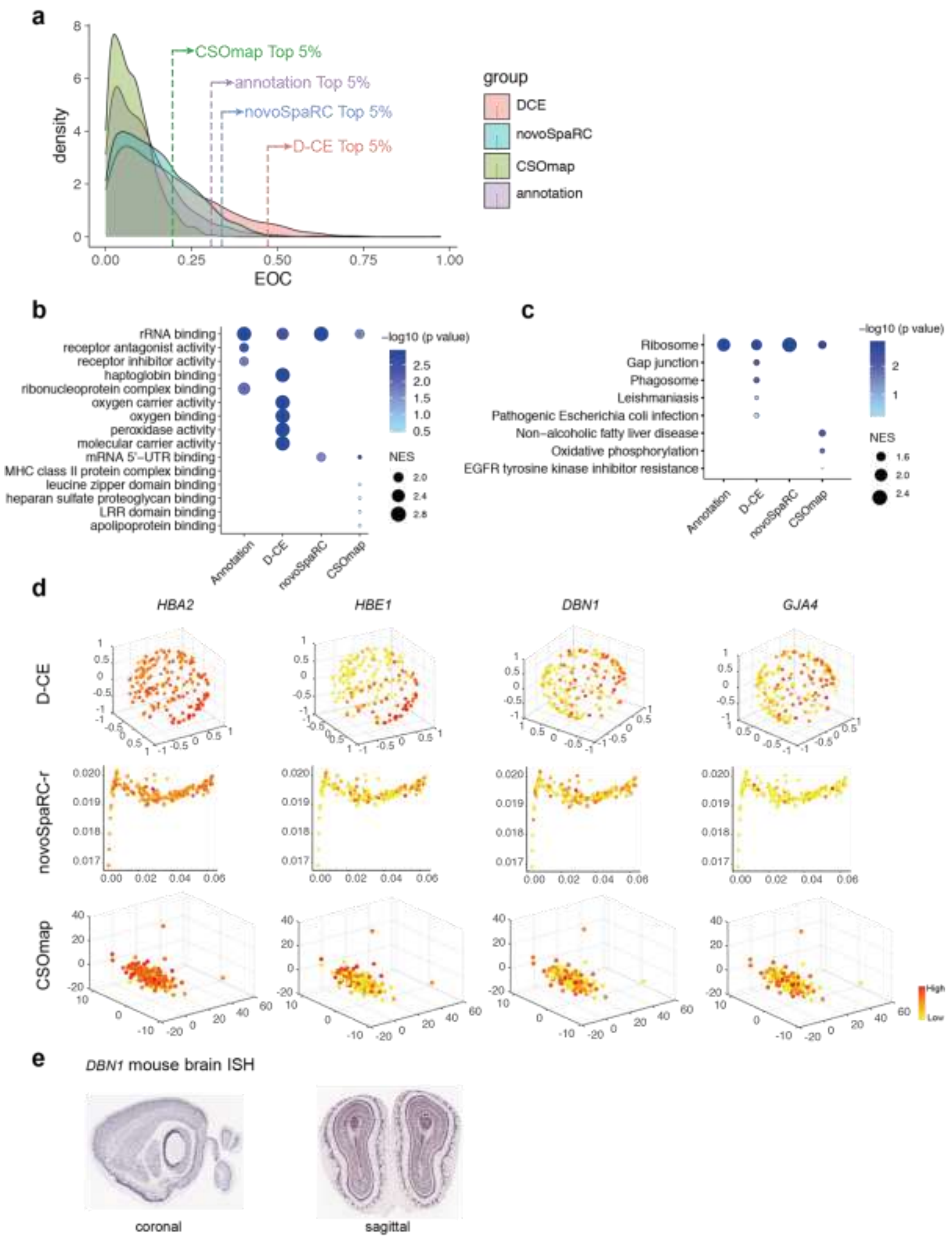


**Supplementary Figure 4. Comparison of genes' EOC to reconstructed sample orders versus annotated spatial domain order in human embryonic cerebral cortex dataset. a,** Density plot of EOC of all genes to the annotated region order or

the 3 methods' reconstructed coordinates for human embryonic frontal lobe data shown in Figure. 2d. The dashed line marks the top 5% EOC of each distribution. **b and c,** GSEA analysis of GO (d) and KEGG (e) enrichment terms of top 5% EOC genes in panel a. **d,** Visualization of the top 2 EOC genes in D-CE enriched oxygen binding (*HBA2* and *HBE1*) and gap junction (*DBN1* and *GJA4*) term. **e,** In situ hybridization for *DBN1* in mouse brain coronal and sagittal (regenerated from https://portal.brain-map.org/).

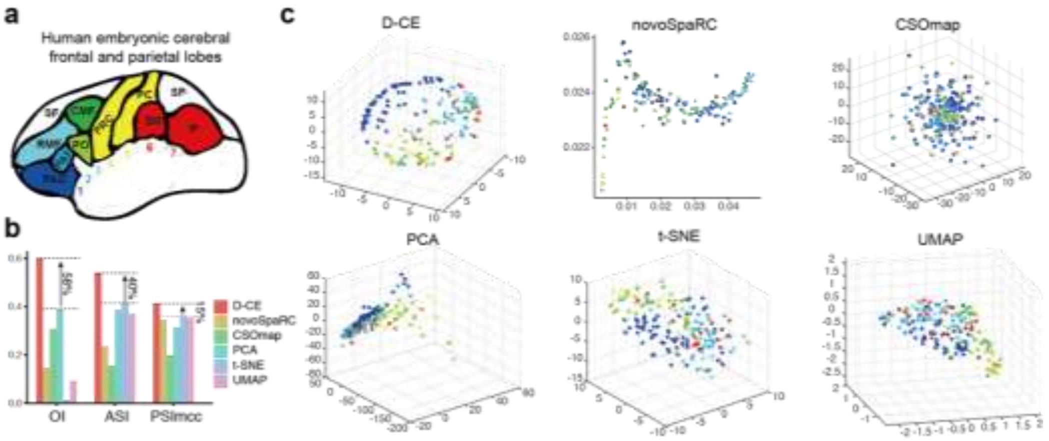


**Supplementary Figure 5. Reconstruction of spatial domain of human embryonic frontal lobe and parietal lobe by D-CE, novoSpaRC, CSOmap, PCA, t-SNE and UMAP. The same layout as Figure 2a to c.** We used sample 22WF_B1 for 3D cell reconstruction of both frontal lobe and parietal lobe, as only this sample contained cells from all brain regions shown in panel a. Due to the small number of cells in PRC and SM, 7 and 2 respectively, we colored them with the same color as the adjacent regions.

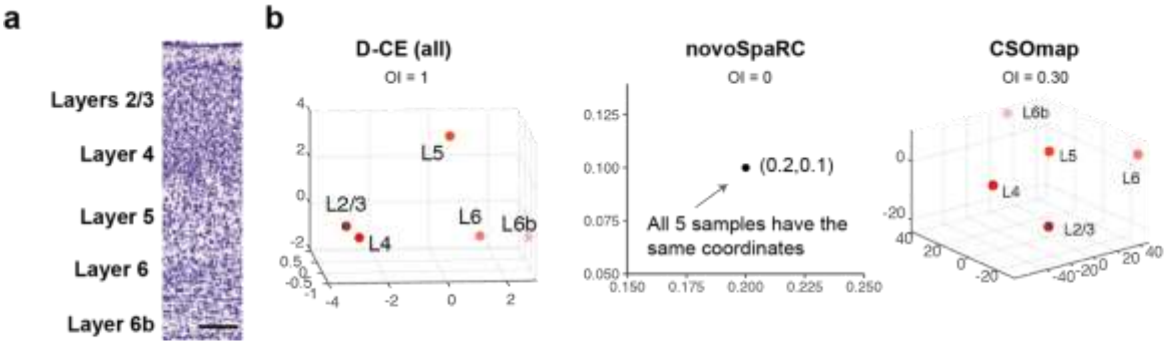

**Supplementary Figure 6. Spatial reconstruction of 5 mouse neocortical layers RNA-seq data with D-CE, novoSpaRC and CSOmap. a,** Cortex stained by Nissl shows the position of 5 mouse neocortical layers (reproduced from Ref. 32). **b,** D-CE (left), novoSpaRC (middle) and CSOmap[42] reconstructed structure Colored from dark red (L2/3) to light red (L6b).

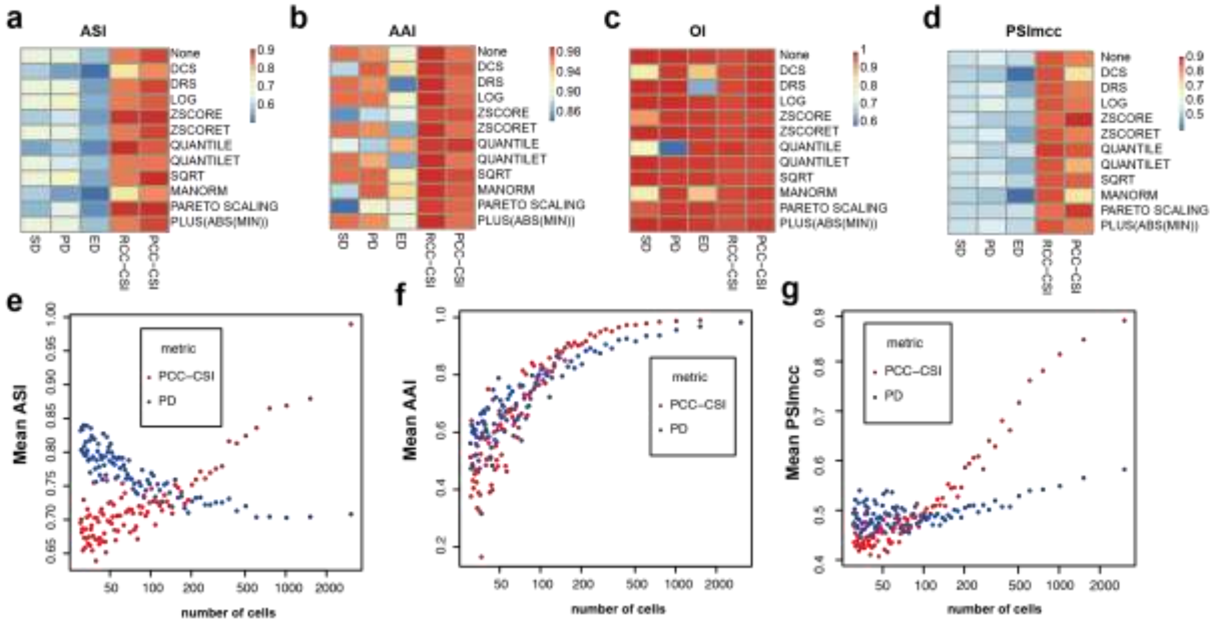


**Supplementary Figure 7. PCC-CSI versus PD for D-CE reconstruction on the fly embryo BDTNP dataset. a** to **d**, ASI, OI (the minimal OI of x, y, and z axis) and PSImcc of spatial reconstruction of the BDTNP dataset using different normalization method and distance/similarity metric. **e** and **g**, The mean ASI and PSImcc of spatial reconstruction of Drosophila embryo BDTNP dataset. Datasets of 1/n (n=2~100) of the BDTNP dataset were generated by randomly selecting samples at each indicated sampling rate, 20 repetitions were made at each sampling rate. The mean ASI and PSImcc of the reconstructed coordinates of 20 repeats by Pearson distance network and PCC-CSI network, respectively, are calculated and visualized.

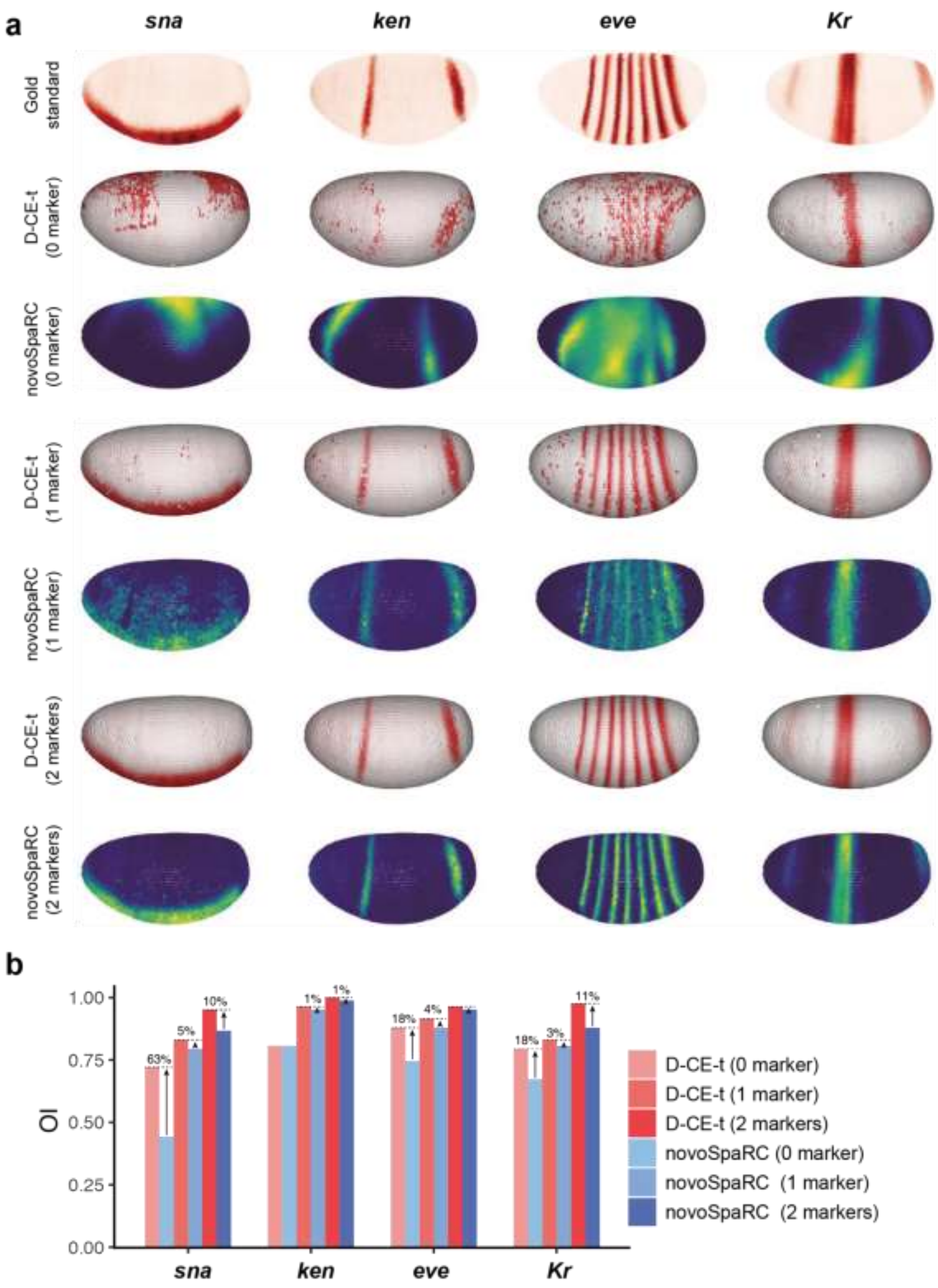


**Supplementary Figure 8. Visualization of the expression patterns of 4 TFs (*sna*, *ken*, *eve* and *Kr*) in embryo structures reconstructed by D-CE-t or novoSpaRC.**

**a,** D-CE-t reconstructed spatial expression pattern of 4 spatial specific genes (sna, ken, eve and Kr) compared to the gold standard image (top), and the novoSpaRc reconstructed structure with 0, 1 and 2 markers. **b,** Barplot of OI. The percentage of improvement by D-CE-t over the novoSparc is labeled on the top of each bar.

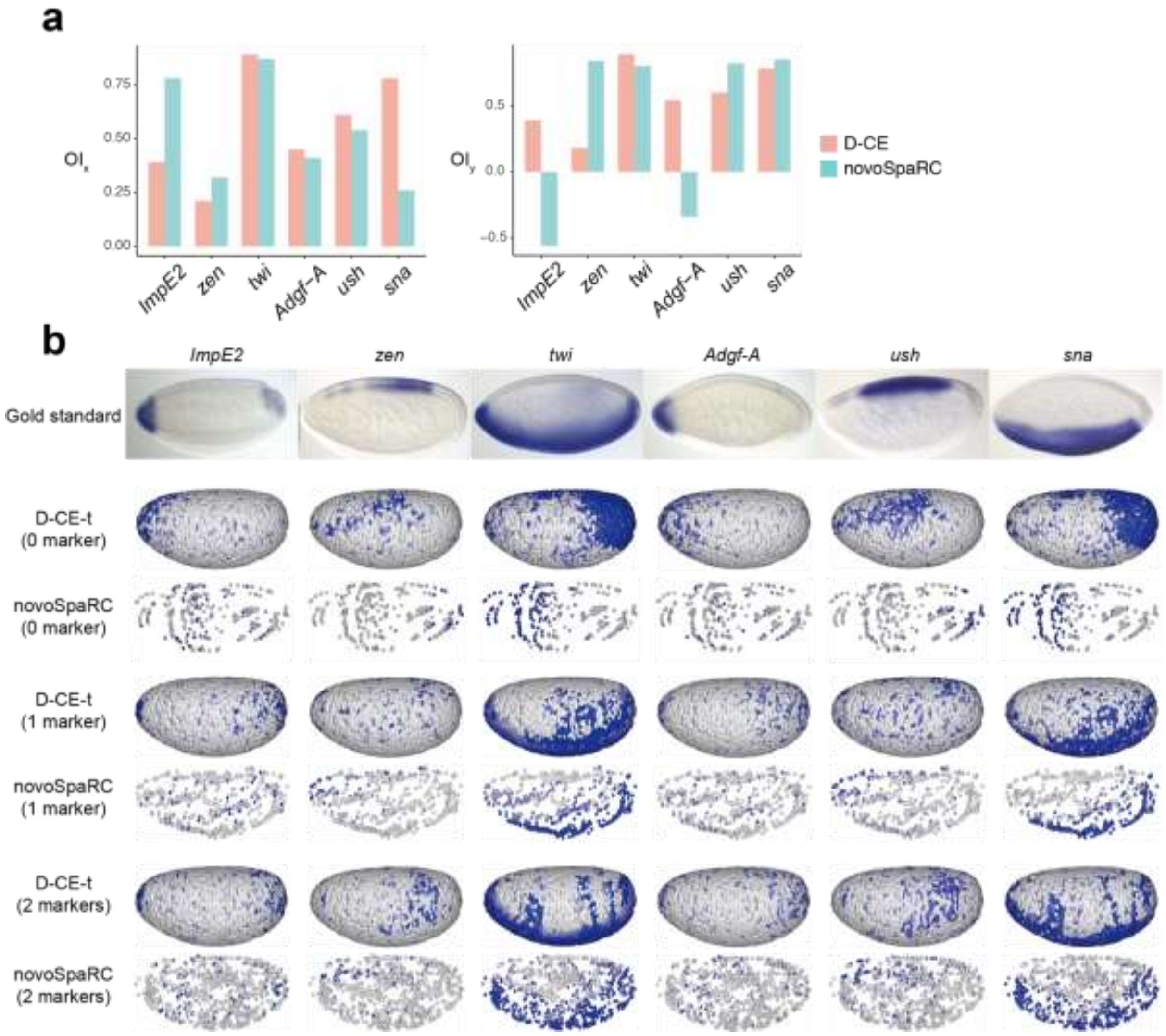


**Supplementary Figure 9. Reconstruction of spatial gene expression patterns in *Drosophila* embryo from an scRNA-seq dataset. a,** Barplot of $OI_x$ (left) and $OI_y$ [42] of 6 spatially expressed genes, including *ImpE2, zen, twi, Adgf-A, ush* and *sna*. OI here is the max OI among all orientations [19]. **b,** Reconstructed expression of 6 spatially expressed genes above, compared to FISH images reproduced from BDGP

dataset[26] (top row). The second to sixth columns are the structures reconstructed by DCE-t and novoSpaRC with 0, 1, 2 markers.

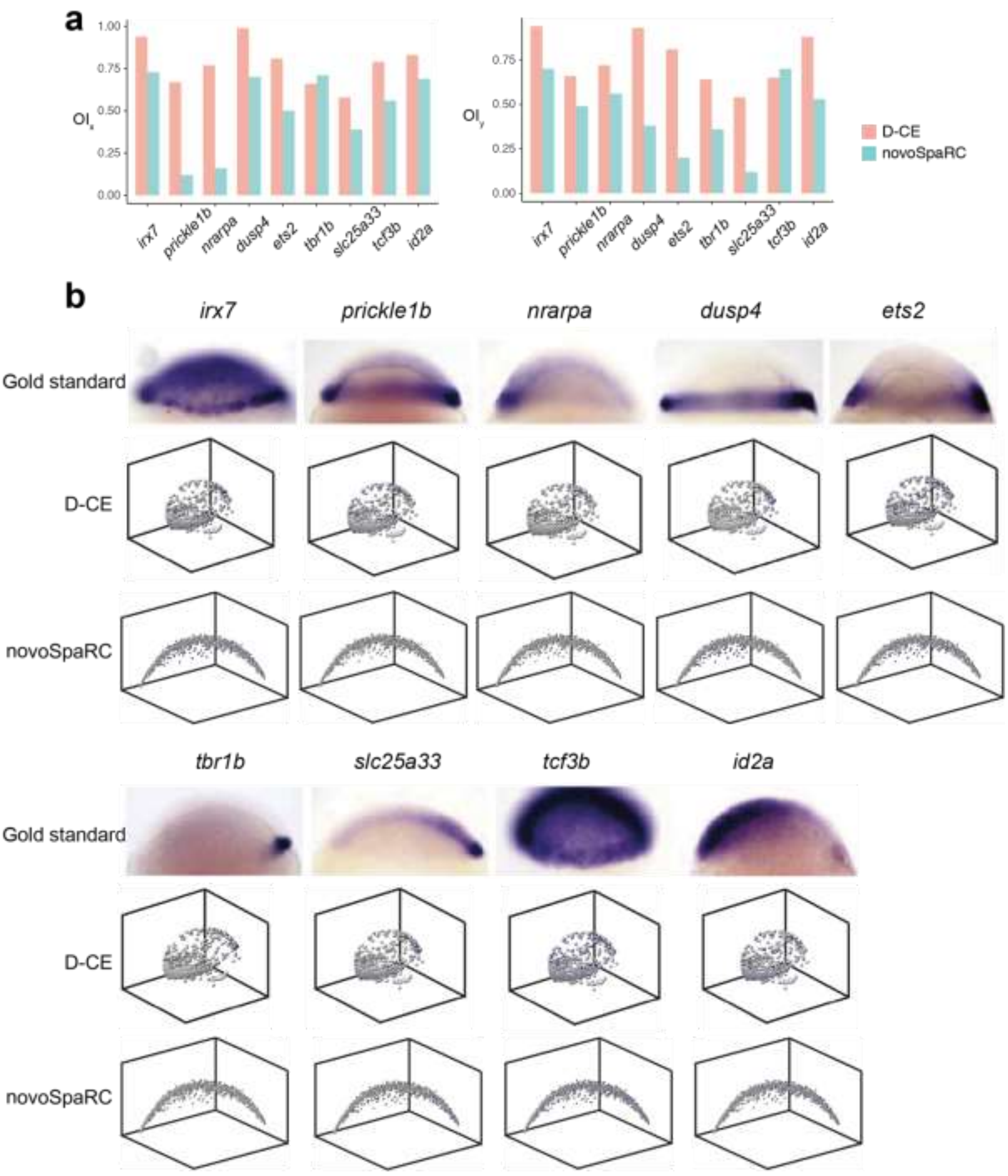


**Supplementary Figure 10. Reconstruction of spatial gene expression patterns using zebrafish embryo from an scRNA-seq data. a,** Barplot of $OI_x$ (left) and $OI_y$

[42] of 9 spatially expressed genes, including *irx7, pickle1b, nrarpa, dusp4, ets2, tbr1b, slc25a33, tcf3b and id2a*. OI here is the max OI among all orientations [19]. **b,** Reconstructed expression of 9 spatially expressed genes above, compared to FISH images reproduced from ref. [27] (top row).

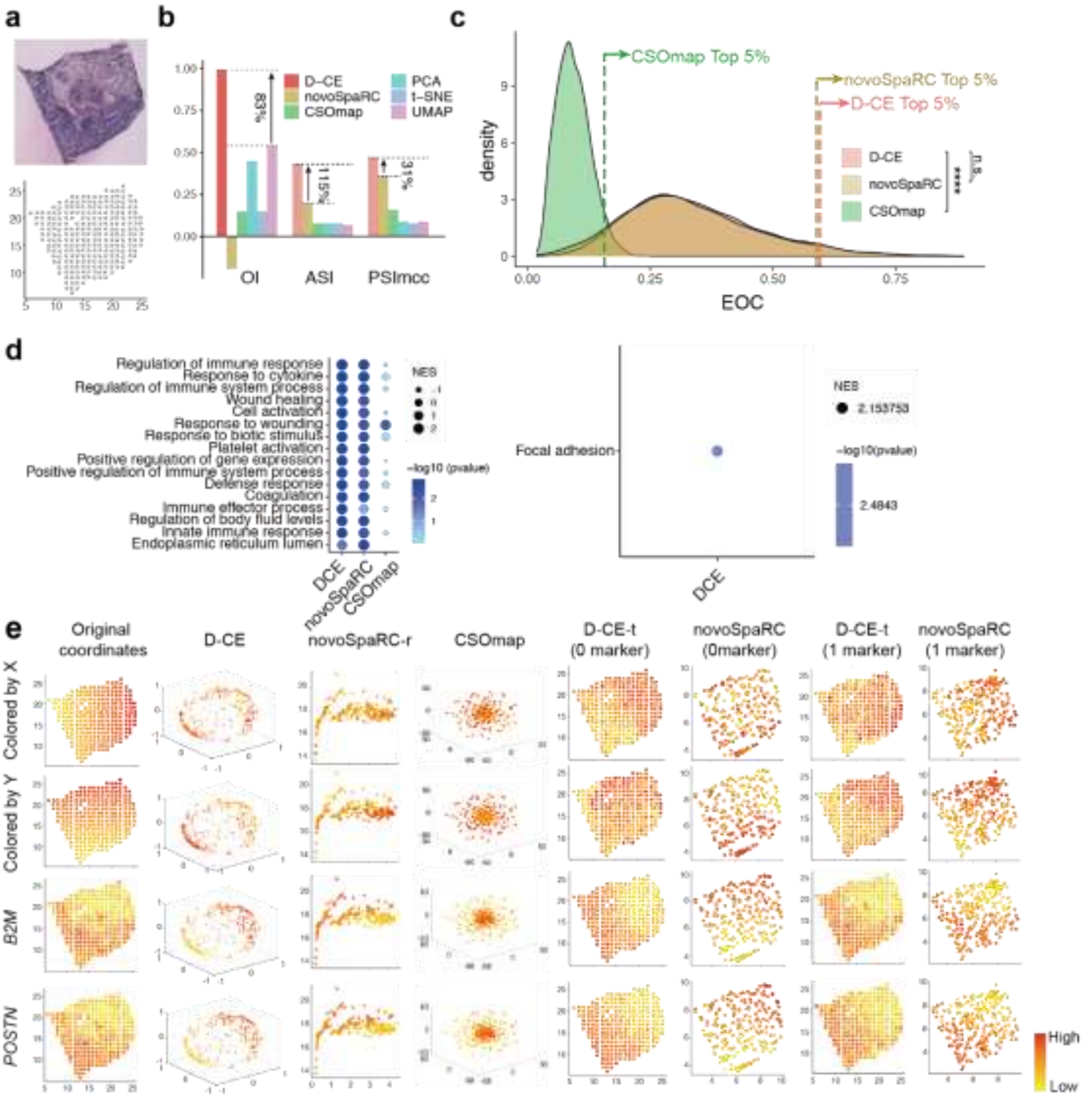


**Supplementary Figure 11. Spatial reconstruction and spatial marker gene detection of human breast cancer spatial transcriptomic dataset with D-CE, novoSpaRC, CSOmap, PCA, t-SNE and UMAP. a,** Human breast cancer tissue staining picture reproduced from Stahl, P. L. *et al***.** (upper panel) and original coordinates of all samples (lower panel ). **b,** Barplot of OI, ASI and PSImcc. **c,** EOC

density plot of D-CE, novoSpaRC and CSOmap. The dashed line indicates the EOC position of top 5% genes in each distribution. Student's t-test was used to compare the difference between D-CE and the other two methods. **d,** GSEA analysis of GO (left) and KEGG [42] enrichment terms of top 5% EOC genes in panel c. Only top EOC genes of D-CE enriched in one KEGG term, "focal adhesion". **e,** Original coordinates, D-CE, novoSpaRC-r and CSOmap without marker and template fitting, D-CE-t with 0 marker, novoSpaRC with 0 marker and template fitting, D-CE-t with 1 marker, and novoSpaRC with 1 marker and template fitting from the first to the eighth column reconstructed coordinates colored according to the X axis, Y axis, and the top two markers' (*B2M* and *POSTN*) expression level from the first to the fourth row, respectively.

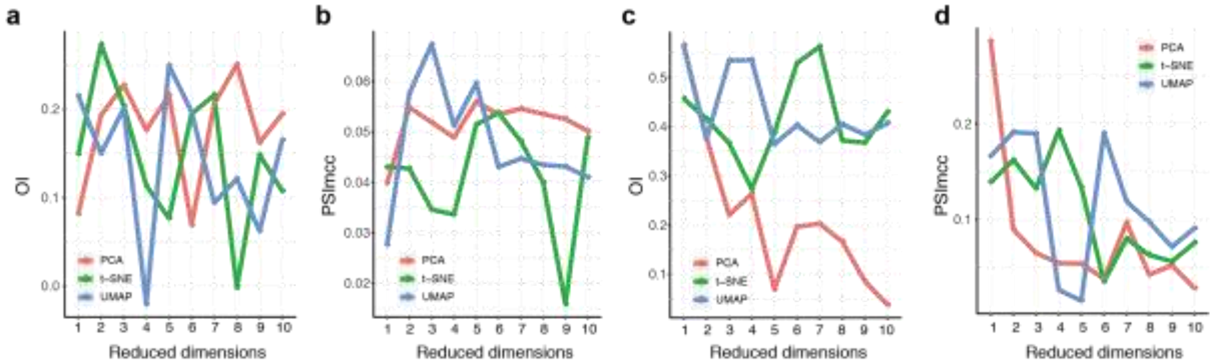


**Supplementary Figure 12.** Mean OI and PSImcc between the original coordinates of spatial transcriptome data and the first to tenth reduced dimensions of PCA, t-SNE and UMAP on olfactory bulb (a and b) and cancerous prostate (c and d) datasets, where OIs of D-CE reconstruction are 0.51 and 0.99, and PSImccs of D-CE reconstruction are 0.46 and 0.58, respectively.

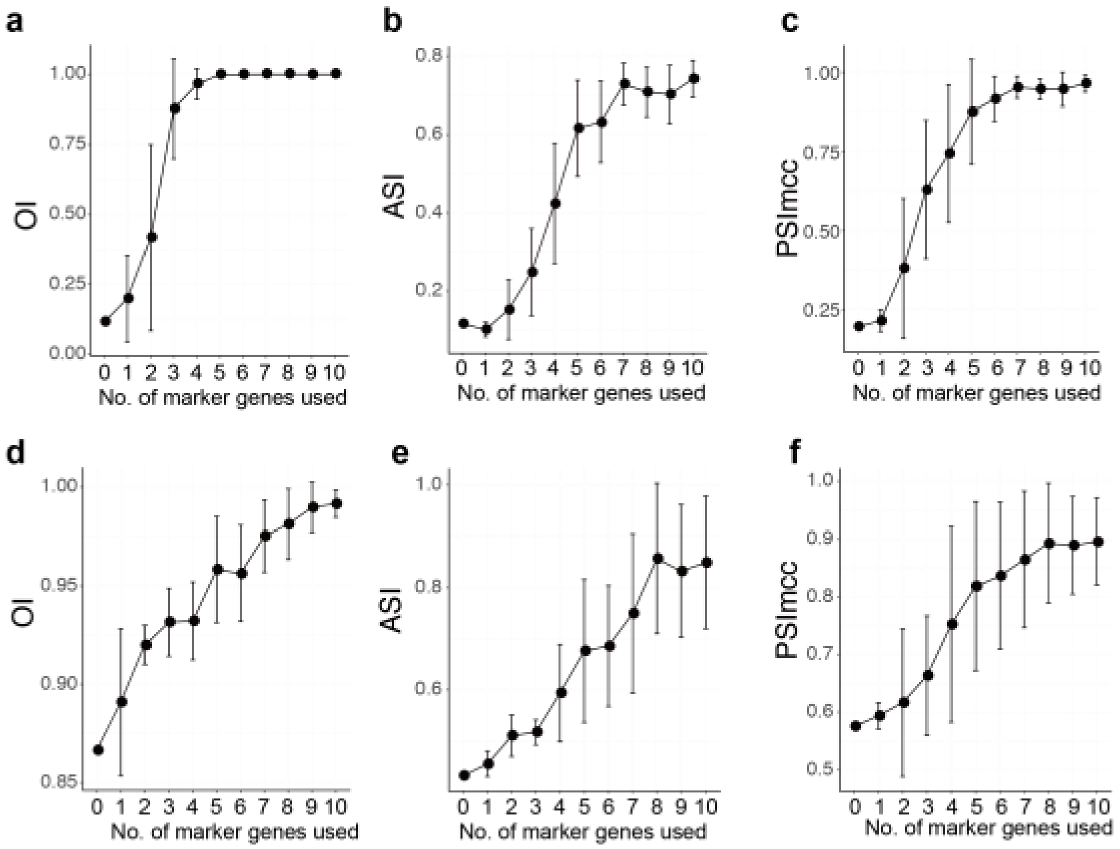


**Supplementary Figure 13. The overall OI, ASI and PSImcc between original sample location and D-CE reconstructed spatial location with increasing of marker genes used. a** to **c,** OI, ASI and PSImcc based on the olfactory bulb dataset. **d** to **f,** OI, ASI and PSImcc based on cancerous prostate dataset.

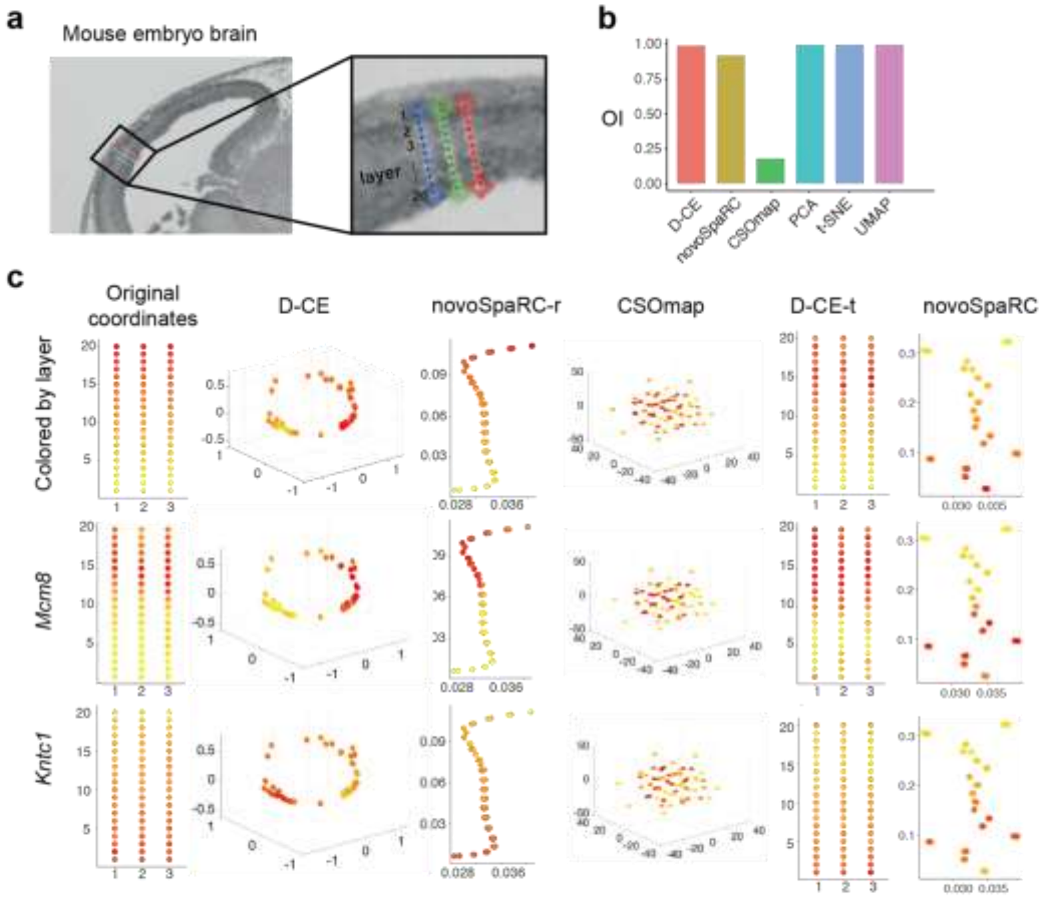


**Supplementary Figure 14. Reconstruction of mouse embryo brain ISH image derived gene expression spatial order with D-CE, novoSpaRC, CSOmap, PCA, t-SNE and UMAP. a,** illustration of spatial orders of ISH image derived samples (bins) along 3 radial lines across E14.5 mouse cerebral cortex layers. The 3 radial lines were manually placed across mouse cerebral cortex. Each line was than scaled into 20 bins.[45] **b,** OI barplot of D-CE, novoSpaRC-r and CSOmap. **c,** Original coordinates, D-CE, novoSpaRC and CSOmap without marker and template fitting, D-CE-t with 0 marker and novoSpaRC with 0 marker from the first to the sixth column reconstructed coordinates colored according to the layers, and the top two markers' (Mcm8 and Kntc1) expression level from the first to the third row respectively.

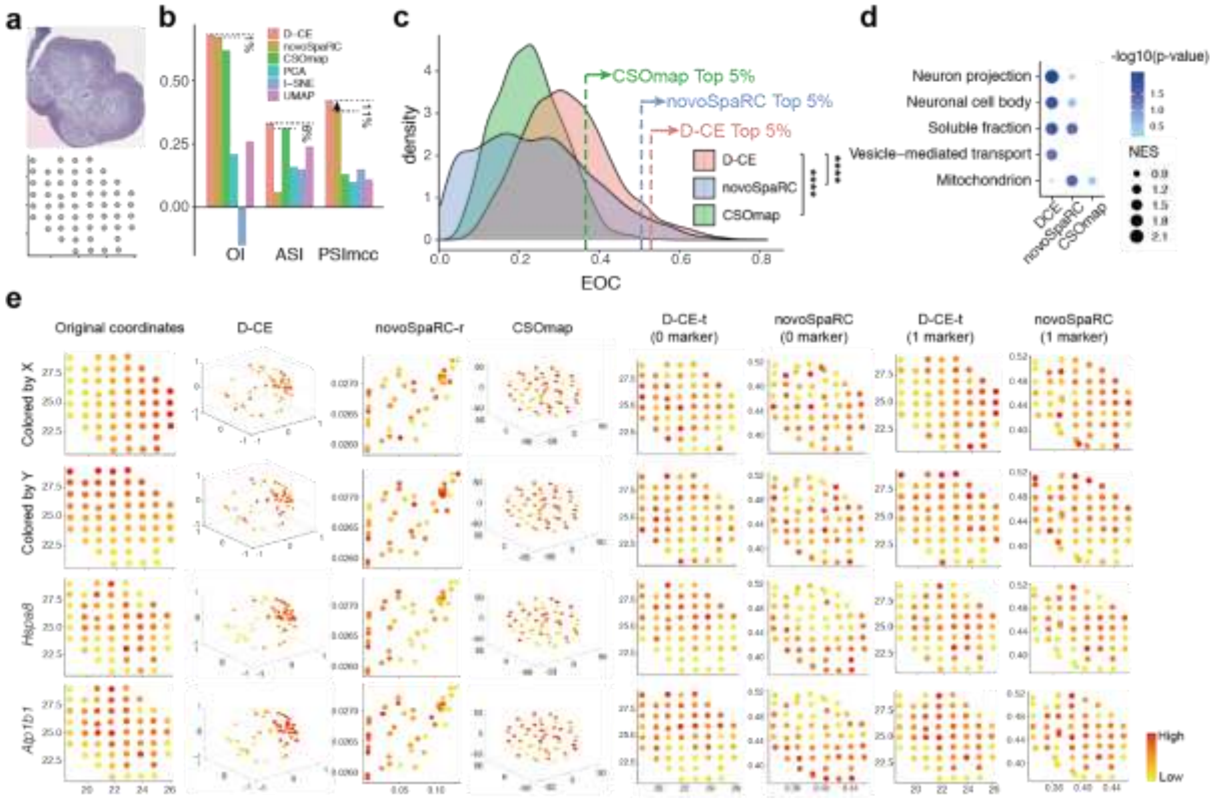


**Supplementary Figure 15. Spatial reconstruction and spatial marker gene detection of mouse spinal cord tissue spatial transcriptomic dataset with D-CE, novoSpaRC, CSOmap, PCA, t-SNE and UMAP. a,** Mouse spinal cord tissue staining picture reproduced from Silas Maniatis *et al.* (upper panel) and original coordinates of all samples (lower panel). **b,** Barplot of OI, ASI and PSImcc. **c,** EOC density plot of D-CE, novoSpaRC and CSOmap. The dashed line indicates the EOC position of top 5% genes in each distribution. Student's T-test was used to compare the difference between D-CE and the other two methods. **d,** GSEA analysis of GO enrichment terms of top 5% EOC genes in panel c. No KEGG term enriched in all three methods. **e,** Original coordinates, D-CE, novoSpaRC-r, CSOmap, D-CE-t with 0 marker, novoSpaRC with 0 marker and template fitting, D-CE-t with 1 marker, and novoSpaRC with 1 marker and template fitting from the first to the eighth column reconstructed coordinates colored according to the X axis, Y axis, and the top two

markers' (Hspa8 and Atp1b1) expression level from the first to the fourth row respectively.

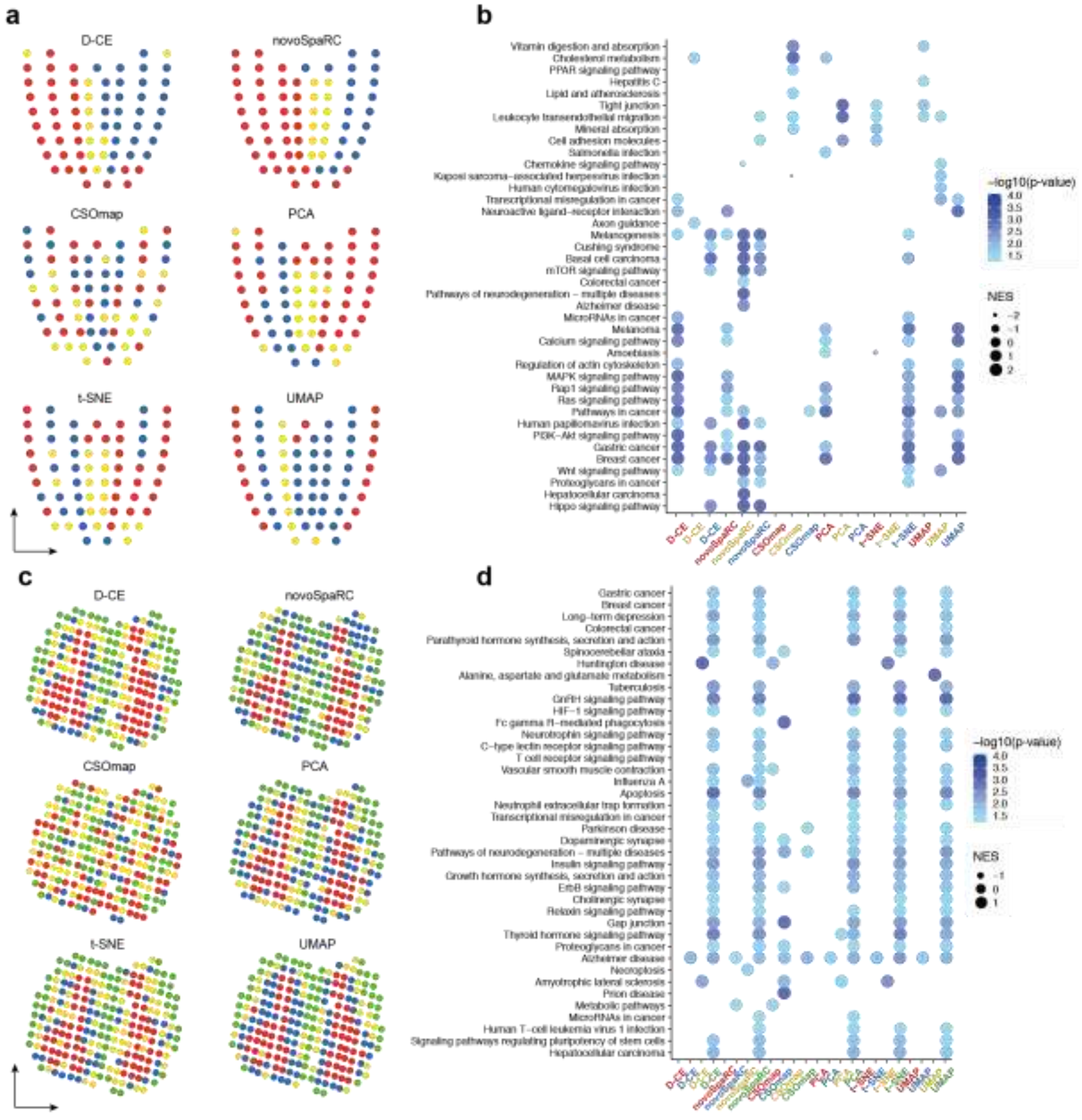


**Supplementary Figure 16. Spatial domain cluster of mouse Geo-seq and olfactory bulb data. a, b,** Same as Figure 6 c and d but for K-means (k = 3). **c and d,** Same as Figure 6c and d but for K-means (k = 4).

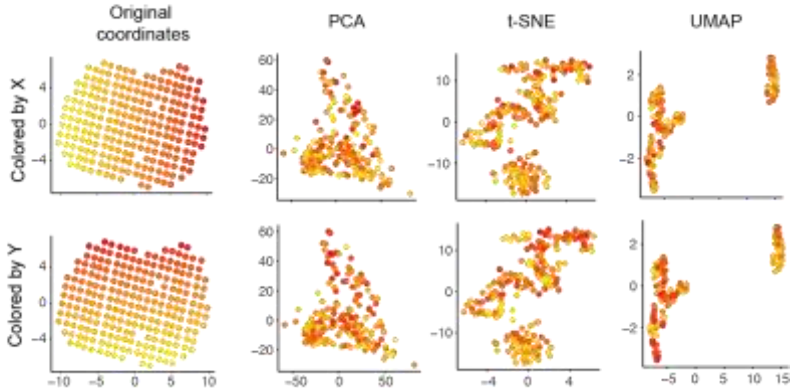


**Supplementary Figure 17. Original coordinates and PCA, t-SNA and UMAP reconstructed structure of olfactory bulb data colored according to the X and Y axis, as in Figure 5.**

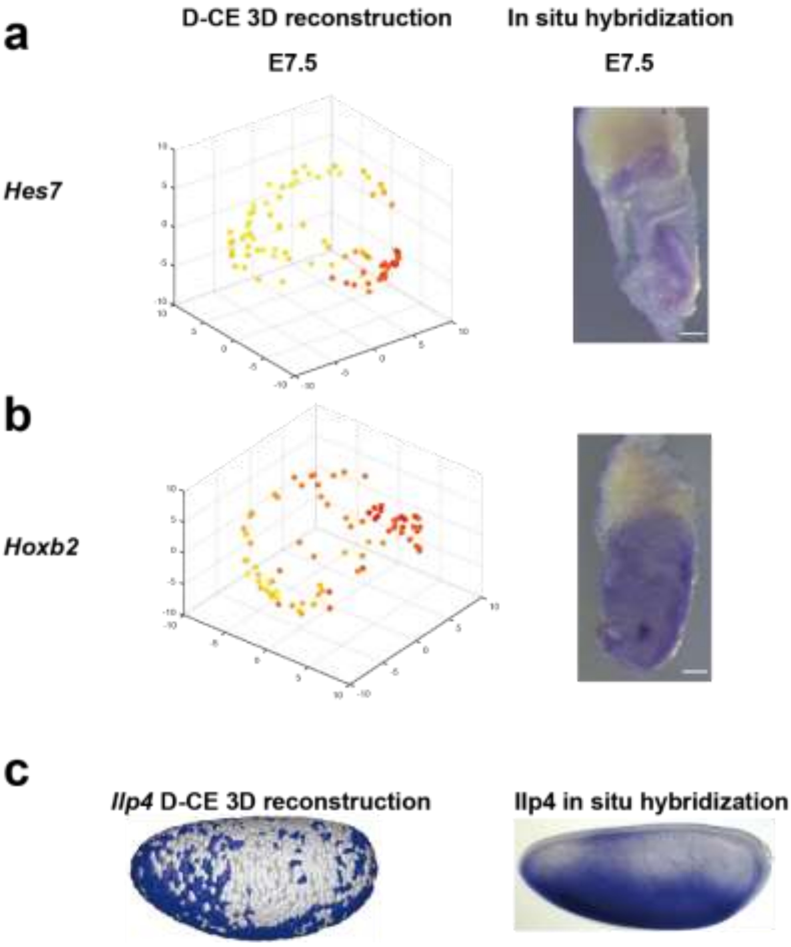

**Supplementary Figure 18. Example of D-CE self-generated spatial markers in mouse and drosophila embryo datasets. a, b**, Visualization of a self-generated spatial marker gene from mouse E7.5 embryo datasets (left panel) and whole mount in situ hybridization experiment validation of the spatial gene expression pattern. Scale bar, 100 μm. **c**, Visualization of a self-generated spatial marker gene from drosophila embryo datasets (left panel) and independent experimental validation in drosophila embryo in situ hybridization (lower panel, regenerated from https://insitu.fruitfly.org/cgi-bin/ex/insitu.pl).

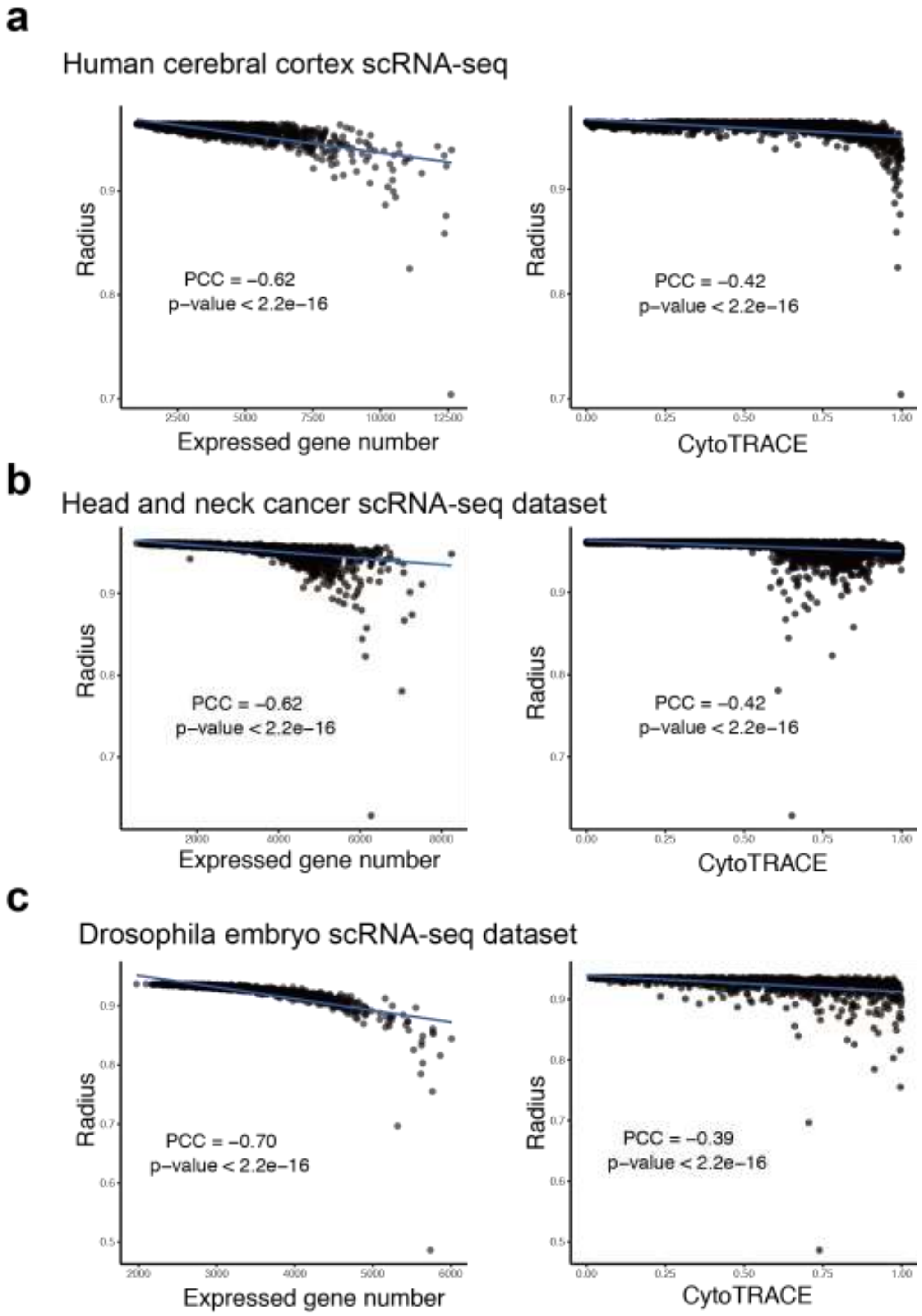


**Supplementary Figure 19. D-CE radius versus number of genes detected/expressed in single cells (left) and CytoTRACE score [42] in three scRNA-seq datasets. a,** human cerebral cortex scRNA-seq. **b,** head and neck cancer scRNA-seq. **c,** Drosophila embryo scRNAseq datasets.

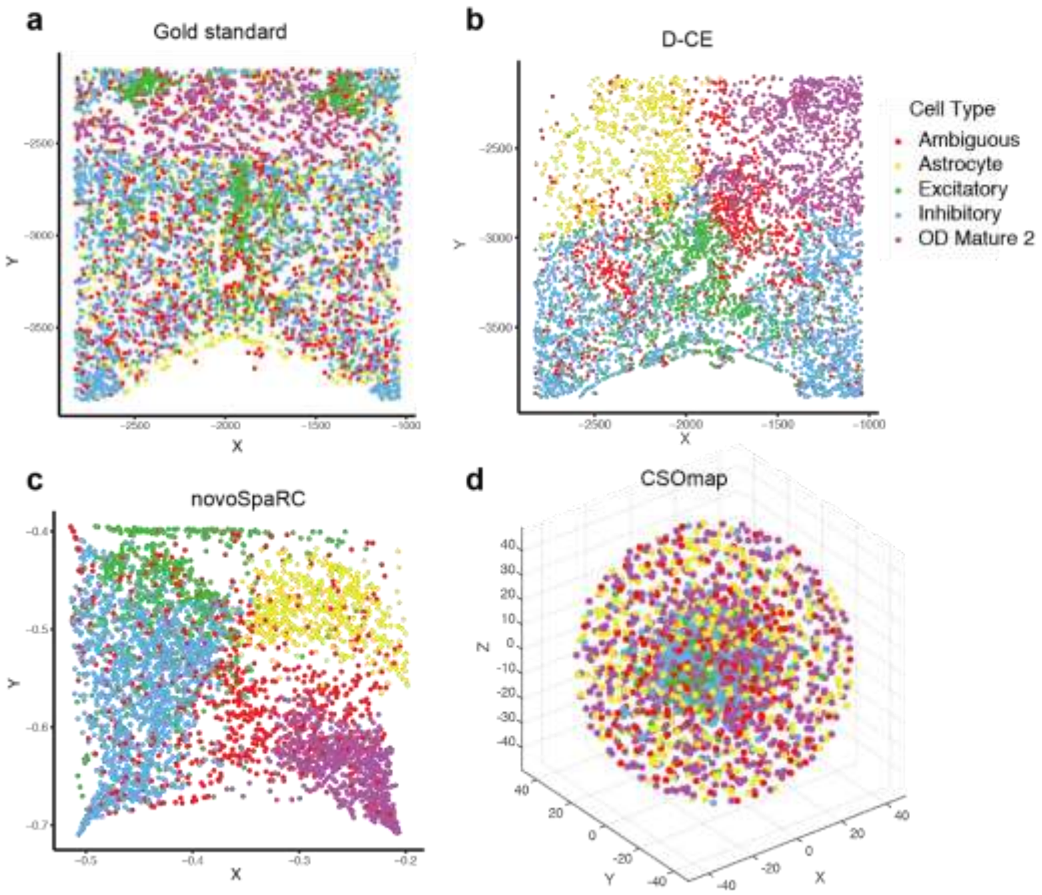


**Supplementary Figure 20. Spatial reconstruction of mouse hypothalamic preoptic region with MERFISH (multiplexed error robust fluorescence in situ hybridization) data**. **a**. The original layout of an example MERFISH slice. **b**, **c** and **d**, Template-fitted reconstructed coordinates of D-CE (b), novoSpaRC (c) and CSOmap (d) of a typical slide. All 161 genes were used here for D-CE, novoSpaRC and CSOmap. Only the top 5 most abundant cell types are visualized. Therefore, not all coordinates are filled out by D-CE.

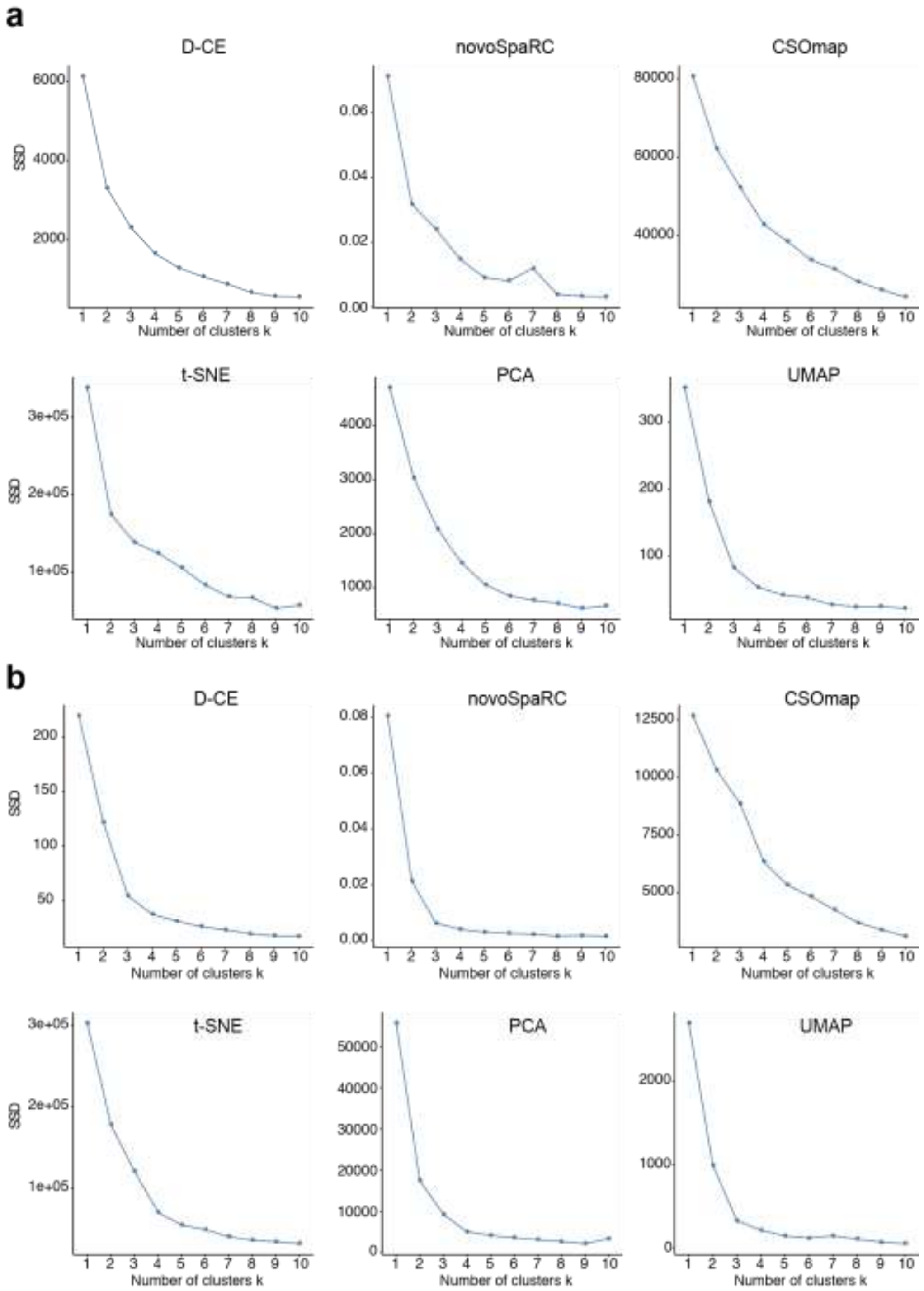


**Supplementary Figure 21. Optimal number of clusters for mouse embryo Geo-seq (a) and olfactory bulb data.** Optimal number of clusters is determined by the inflex point on the total within cluster Sum of Squared Distance (SSD) versus k of the k-means clustering based on reconstructed coordinates.
